# Ga‐on‐In Substitution with Zn Vacancies in Zn_3_In_2_S_6_ Induces Electron–Hole Asymmetry and In─O Bond Weakening for Coupled Two‐Electron Oxygen Reduction and H_2_O_2_ Stabilization

**DOI:** 10.1002/adma.202522831

**Published:** 2026-03-19

**Authors:** Xiaowen Ruan, Chunsheng Ding, Dongxu Jiao, Jing Leng, Minghua Xu, Bonan Li, Zhipeng Yu, Xiaoqiang Cui, Jimmy C. Yu, Yongfa Zhu, Sai Kishore Ravi

**Affiliations:** ^1^ School of Energy and Environment City University of Hong Kong Kowloon Hong Kong SAR China; ^2^ College of Chemistry Chemical Engineering and Resource Utilization Northeast Forestry University Harbin China; ^3^ State Key Laboratory of Chemical Reaction Dynamics Dalian Institute of Chemical Physics Chinese Academy of Sciences Dalian China; ^4^ School of Materials Science and Engineering Key Laboratory of Automobile Materials of MOE Jilin University Changchun China; ^5^ International Iberian Nanotechnology Laboratory (INL) Avenida Mestre Jose Veiga Braga Portugal; ^6^ Department of Chemistry Chinese University of Hong Kong Shatin Hong Kong China; ^7^ Department of Chemistry Tsinghua University Beijing China

**Keywords:** artificial photosynthesis, Ga‐on‐In substitution, H_2_O_2_ Production, Zn_3_In_2_S_6_, Zn Vacancy

## Abstract

Artificial photosynthesis of H_2_O_2_ offers a sustainable route to decentralized chemical production, yet remains limited by sluggish oxygen reduction kinetics, rapid charge recombination, and undesired decomposition of H_2_O_2_ on catalyst active sites. Herein, we report a Zn_3_In_2_S_6_ catalyst (Ga‐ZvIS) featuring Ga‐on‐In substitution and Zn vacancies that together establish electron–hole asymmetry and weaken In**─**O bonding. Ga substitution on In sites lowers the In‐5p‐band center level and reduces H_2_O_2_ adsorption strength, thereby suppressing surface decomposition, while Zn vacancies serve as hole‐localized domains that accelerate isopropanol oxidation and furnish the protons required for the two‐electron oxygen reduction reaction (2e^−^ ORR). This site‐specific dopant–defect interplay produces energetically differentiated electron‐ and hole‐dominated regions, promotes directional charge migration, and sustains the 2e^−^ ORR pathway. The optimized catalyst exhibits a H_2_O_2_ production rate of 187.8 µmol g^−^^1^ min^−^^1^ in O_2_‐saturated aqueous isopropanol, outperforming most reported photocatalysts. Kelvin probe force microscopy and femtosecond transient absorption spectroscopy confirm efficient carrier separation consistent with the built‐in electrostatic potential arising from electron–hole asymmetry, while DFT calculations reveal favorable O_2_ adsorption and weakened H_2_O_2_ binding on Ga–In sites. A proof‐of‐concept continuous‐flow photoreactor further demonstrates in situ Fenton‐assisted oxidation of organic contaminants, validating the practical utilization of the photosynthesized H_2_O_2_.

## Graphical abstract

Ga‐on‐In substitution coupled with Zn vacancies in Zn_3_In_2_S_6_ creates a dopantdefect synergy that weakens InO bonding and induces electronhole asymmetry. The resulting spatial charge separation directs the two‐electron oxygen reduction pathway, enabling efficient artificial photosynthesis of H_2_O_2_ with a production rate of 187.8 µmol g^−1^ min^−1^.

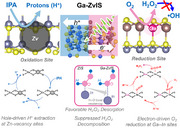

## 1 Introduction

Hydrogen peroxide (H_2_O_2_) is a versatile and important green oxidant with broad applications in organic synthesis, disinfection, and clean fuel production [1, 2, 3, 4]. Conventional industrial production, dominated by the anthraquinone process, relies on energy‐intensive, multistep operations under harsh conditions and generates substantial chemical waste [5, 6, 7]. These limitations have motivated the development of alternative, environmentally benign routes. Artificial photosynthesis of H_2_O_2_, which directly converts H_2_O and O_2_ using solar energy, has emerged as a promising approach owing to its mild operating conditions, high product purity, and sustainability [8, 9, 10].

Photocatalytic H_2_O_2_ production generally proceeds through two coupled half‐reactions: the two‐electron oxygen reduction (O_2_ + 2H^+^ + 2e^−^ → H_2_O_2_, 0.68 V versus NHE) and the water oxidation (2H_2_O + 2h^+^→ H_2_O_2_ + 2H^+^, 1.78 V versus NHE) [11, 12]. A central challenge lies in designing photocatalysts that exhibit high selectivity toward the two‐electron O_2_ reduction pathway while maintaining product stability [13, 14]. Although considerable progress has been made in enhancing H_2_O_2_ generation rates, parasitic decomposition of H_2_O_2_ on catalyst surfaces remains a major bottleneck. Strong chemisorption and side reactions can induce O─O bond cleavage, deactivate active sites, and hinder subsequent O_2_ reduction [15, 16]. Addressing this issue requires precise control over both bulk electronic structure and surface adsorption properties: efficient charge separation and transport must be coupled with moderated H_2_O_2_ binding and facile desorption. Coordinated optimization of these bulk–surface characteristics is therefore critical for simultaneously improving H_2_O_2_ selectivity, yield, and durability [17, 18].

**SCHEME 1:**
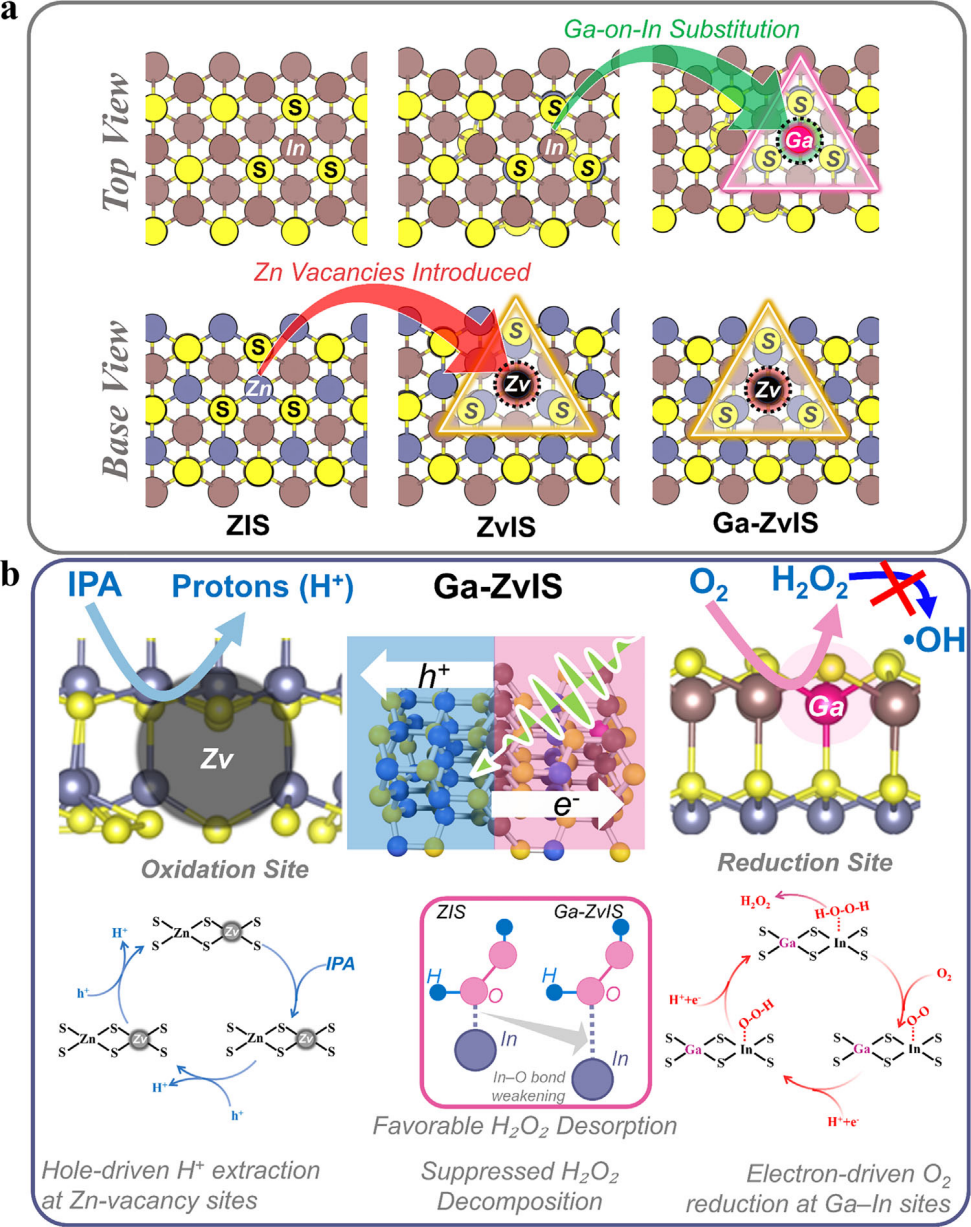
Schematic illustration of catalyst design and reaction mechanism (a) Structural design of Ga‐on‐In substituted and Zn‐vacancy‐engineered Zn_3_In_2_S_6_ (Ga‐ZvIS), illustrating the evolution from pristine ZIS to electron‐ and hole‐localized active domains. (b) Photocatalytic H_2_O_2_ synthesis mechanism over Ga‐ZvIS, showing hole‐driven proton extraction at Zn‐vacancy sites and electron‐guided O_2_ reduction at Ga–In sites, which collectively enhance H_2_O_2_ generation and stability.

Ternary sulfides, Zn_m_In_2_S_m+3_ (m = 1–3), a family of n‐type semiconductors, have attracted growing interest in photocatalysis due to their favorable band structures, strong redox potentials, and photoelectric properties [19, 20, 21]. Among them, Zn_3_In_2_S_6_ possesses a positive valence band (+1.67 eV) and a negative conduction band (−0.49 eV), providing sufficient thermodynamic driving force for both oxidation and reduction reactions. However, pristine Zn_3_In_2_S_6_ suffers from pronounced bulk charge recombination, limited carrier mobility, and insufficient surface‐active sites (Scheme 1a). Defect engineering has been widely adopted to mitigate these limitations by introducing shallow trap states that enhance exciton dissociation and charge separation reactions [22, 23, 24], but excessive defect concentrations can generate deep recombination centers and compromise lattice stability.

Heteroatom doping offers an alternative strategy to modulate electronic structure and improve carrier transport. In particular, Ga doping can hybridize with In orbitals, enhance electron mobility, and tune the conduction‐band position through orbital coupling and band dispersion [25]. Owing to its filled electronic configuration, Ga incorporation suppresses the formation of mid‐gap states and facilitates efficient charge separation [26, 27, 28]. Nevertheless, single‐parameter modification remains insufficient to simultaneously regulate electron and hole dynamics, motivating the integration of dopant engineering with vacancy formation to achieve balanced charge separation, redox activity, and product stability.

Here, we report a Ga‐on‐In and Zn‐vacancy co‐modified Zn_3_In_2_S_6_ catalyst (denoted as Ga–ZvIS) designed to establish electron–hole asymmetry and modulate In**─**O bonding (Scheme 1a). Ga substitution lowers the In‐5p‐ band center level and weakens In**─**O interactions, reducing H_2_O_2_ adsorption strength and suppressing its decomposition. Concurrently, Zn vacancies localize photogenerated holes, promote isopropanol oxidation, and supply protons to sustain the two‐electron oxygen reduction reaction (2e^−^ ORR). This site‐specific dopant–defect synergy (Scheme 1b) creates energetically differentiated electron‐ and hole‐dominated regions, enabling directional charge migration and coupled H_2_O_2_ formation and stabilization within a single lattice.

As a result, the optimized Ga–ZvIS catalyst achieves a H_2_O_2_ production rate of 187.8 µmol g^−^^1^ min^−^^1^ in O_2_‐saturated aqueous isopropanol, outperforming most reported photocatalysts. In situ XPS and femtosecond transient absorption spectroscopy reveal enhanced built‐in electrostatic potential, suppressed carrier recombination, and improved directional charge transport upon Ga and Zn‐vacancy incorporation. Kelvin probe force microscopy and in situ FTIR further confirm electron‐rich Ga–In domains favoring ORR and hole‐rich Zn‐vacancy domains promoting water/isopropanol oxidation. H_2_O_2_ decomposition experiments and density functional theory calculations demonstrate that Ga‐on‐In substitution weakens In**─**O bonding and stabilizes H_2_O_2_, while Zn vacancies act as interfacial hole sinks that accelerate oxidation and proton generation. Collectively, this element‐specific dopant–vacancy strategy addresses the long‐standing limitations of single‐phase photocatalysts for artificial H_2_O_2_ photosynthesis.

## 2 Results and Discussion

### 2.1 Synthesis and Characterizations of Catalysts

**FIGURE 1:**
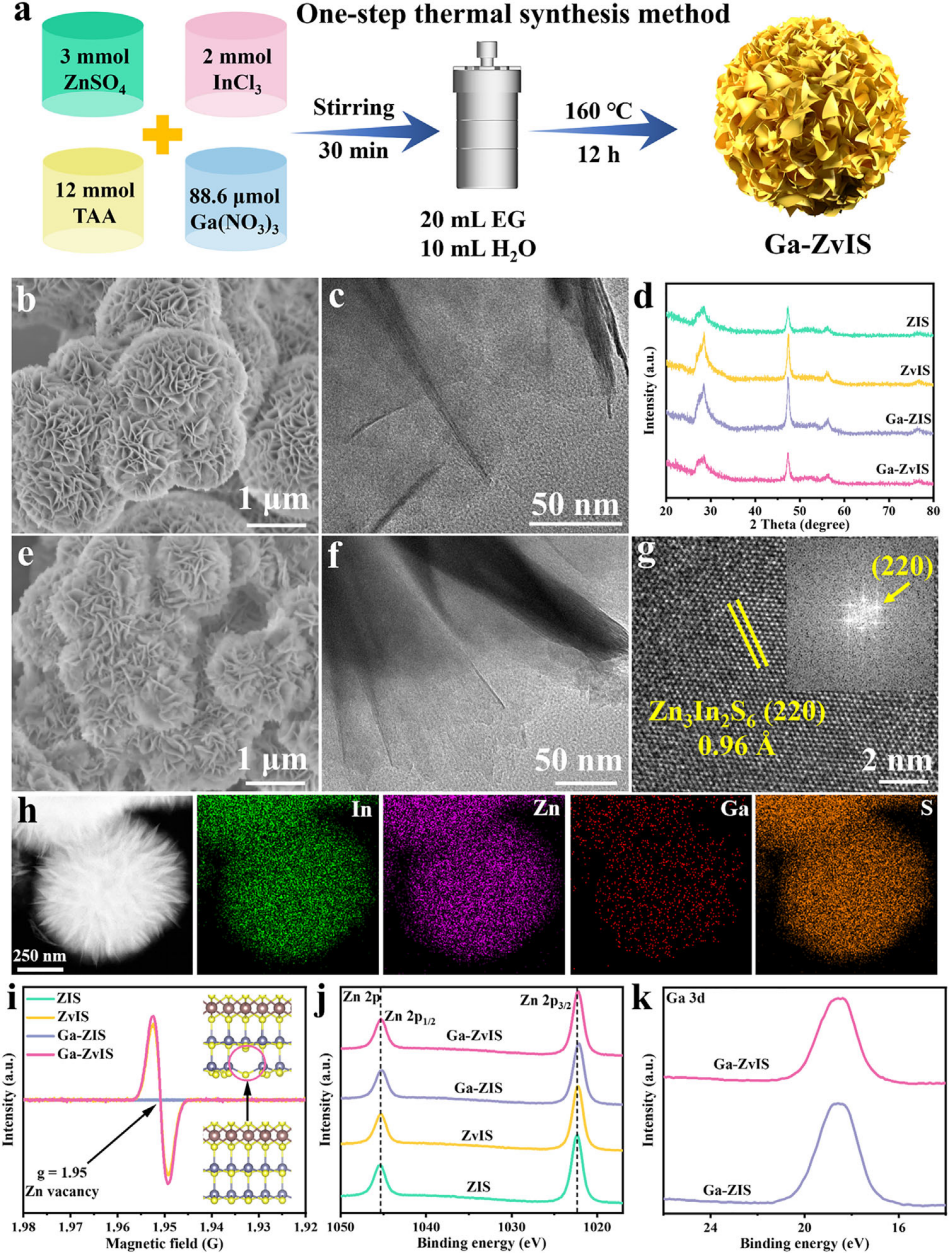
Synthesis route and structural characterization (a) Schematic diagram of the synthetic route toward Ga‐ZvIS. (b) SEM and (c) TEM image of pristine ZIS. (d) XRD patterns of catalysts. (e) SEM and (f) TEM image of Ga‐ZvIS catalyst. (g) HRTEM and (h) Elemental mapping images of Ga‐ZvIS. (i) ESR and XPS spectra of (j) Zn 2p and (k) Ga 3d of catalysts.

Figure 1a displays the synthesis route of designed Ga‐ZvIS catalyst. Specifically, the synthesis of Ga‐doped ZIS with Zn vacancies was achieved via a one‐step hydrothermal/solvent‐thermal process: The solvent effect can significantly influence the crystallization process, thereby determining the morphology and defect distribution of the material. Ethylene glycol (EG) is commonly used in solvothermal methods to adjust the composition and morphology of synthesized materials. The special coordination relationship between EG and Zn^2+^ can produce both a high desolvation energy and a decrease in the Zn^2+^ diffusion coefficient, thereby increasing the nucleation energy barrier and potentially inducing the formation of Zn vacancies [29, 30]. Scanning electron microscopy (SEM) and transmission electron microscopy (TEM) were used to investigate the morphology and structure of pristine ZIS and modified ZIS catalyst. Compared with pristine ZIS, the as‐synthesized ZvIS, Ga‐ZIS, and optimized Ga‐ZvIS catalyst exhibit a similar sea urchin‐like structure composed of abundant nanosheets (Figure 1b–f, Figures S1 and S2). This indicates that introducing Zn vacancies and Ga dopant preserves the basic ZIS framework, as confirmed by XRD patterns showing no detectable impurity peaks (Figure 1d). The HRTEM image reveals crystal facets with spacings of 0.96 Å, corresponding to the (220) plane of the hexagonal ZIS phase, and the structure of Ga‐ZvIS is further validated by fast Fourier transform (FFT) analysis (Figure 1g, Figure S3). Elemental mapping images exhibit the homogeneous distribution of In, Ga, Zn, and S throughout the Ga‐ZvIS catalyst (Figure 1h). To verify the coexistence of Zn vacancies and Ga doping, electron spin resonance (ESR) spectra were recorded. A characteristic signal with a g‐value of 1.95 is detected in both ZvIS and Ga‐ZvIS catalysts, consistent with unpaired electrons localized at sulfur‐coordinated Zn‐vacancy sites, typical for II–VI sulfides, at the same time, no signal for the S vacancy was detected (Figure 1i, Figure S4) [31]. The Ga‐ZvIS sample shows comparable ESR intensity to ZvIS, indicating that Ga doping acts cooperatively with Zn vacancies to enhance activity. X‐ray photoelectron spectroscopy (XPS) was employed to investigate the presence of Ga species and the composition of the catalyst. The Zn 2*p* spectra display two distinct peaks at binding energies of 1022.38 and 1045.28 eV in all as‐prepared catalysts, which are ascribed to Zn 2*p*_3/2_ and Zn 2*p*_1/2_ of Zn^2+^, respectively (Figure 1j). The Ga 3*d* spectra exhibit a typical peak at binding energies of 18.4 eV in Ga‐ZIS and Ga‐ZvIS catalyst, which is consistent with that of previous reported research (Figure 1k). Additionally, the In 3d and S 2p spectra of catalysts are provided and confirm that our designed Ga‐ZvIS catalyst is successfully synthesized (Figures S5 and S6).

**FIGURE 2:**
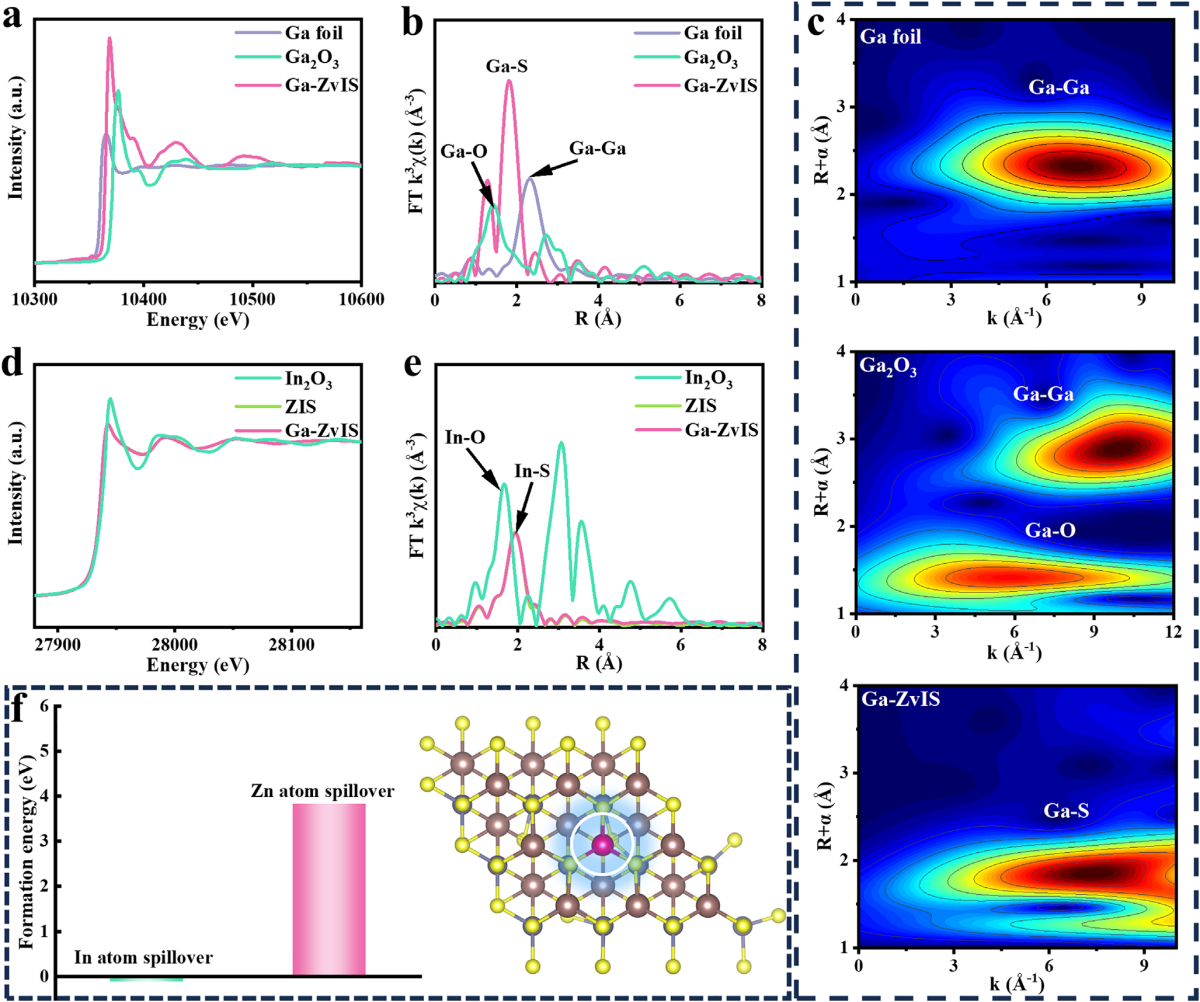
Local structure and coordination environment (a) Normalized Ga K‐edge XANES spectra of Ga foil, Ga_2_O_3,_ and Ga‐ZvIS, (b) Fourier transformed EXAFS spectra of Ga foil, Ga_2_O_3,_ and Ga‐ZvIS. (c) Wavelet transform (WT) of Ga foil, Ga_2_O_3,_ and Ga‐ZvIS. (d) Normalized In K‐edge XANES spectra of In_2_O_3_, ZIS, and Ga‐ZvIS, (e) Fourier transformed EXAFS spectra of In_2_O_3_, ZIS, and Ga‐ZvIS. (f) The energy required for In and Zn atoms to overspill from ZIS, and the positions occupied by Ga atoms.

X‐ray near‐edge absorption spectroscopy (XANES) and extended X‐ray absorption fine structure (EXAFS) analyses were employed to verify the coordination environment and chemical state of Ga in ZvIS (Figure 2a,b, Figure S7). The XANES absorption intensities of Ga in Ga‐ZvIS lie between those of Ga foil and Ga_2_O_3_, indicating that the Ga species in Ga‐ZvIS catalysts carry a positive charge with a valence state ranging from 0 to +3. Further EXAFS analysis revealed a distinctive peak at 1.81 Å for Ga‐ZvIS, markedly distinct from the main peak positions observed in Ga foil and Ga_2_O_3_. This indicates the absence of Ga and Ga_2_O_3_ nanoparticles within Ga‐ZvIS, with elemental Ga present in ZvIS as Ga─S coordination species. Furthermore, wavelet transform contour plots provide additional confirmation of the existence of Ga‐S coordination (Figure 2c). Further XANES and EXAFS analyses were conducted on the In K‐edge of ZIS and Ga‐ZvIS samples (Figure 2d,e, Figures S8 and S9). The results demonstrate near‐identical outcomes for both ZIS and Ga‐ZvIS samples, indicating that Ga incorporation does not disrupt the basic ZIS structure. Theoretical calculations were employed to further validate the presence of Ga at specific sites. By calculating the energy required for Zn and In atoms to be spilled from the original ZIS structure, it was determined that Ga atoms preferentially occupy the sites of In atoms (Figure 2f).

### 2.2 Carrier Transportation and Separation

**FIGURE 3:**
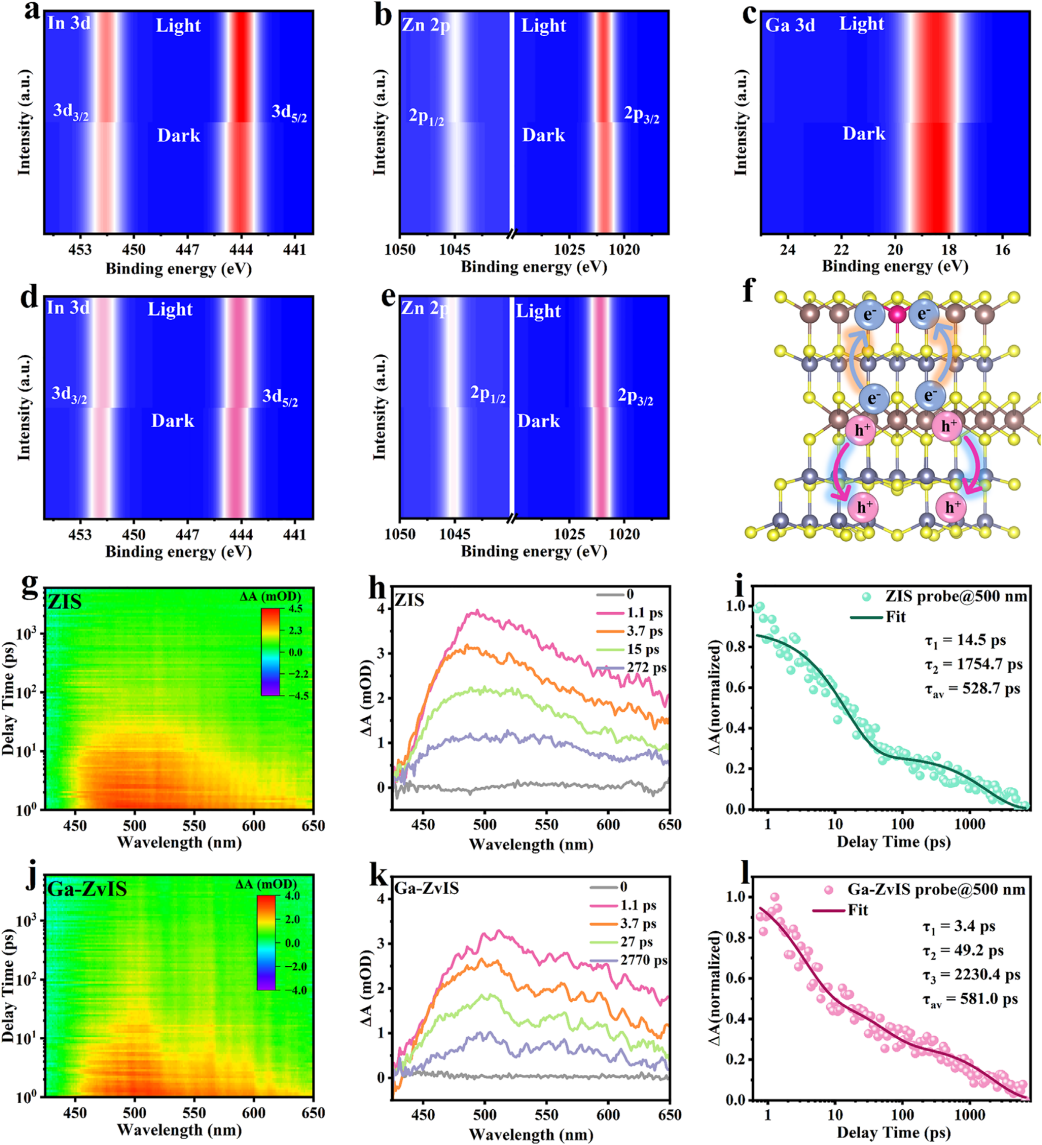
Electronic structure and charge redistribution: In situ XPS spectra of (a) In 3d, (b) Zn 2p, (c) Ga 3d of Ga‐ZvIS catalyst under dark and irradiation. In situ XPS spectra of (d) In 3d and (e) Zn 2p of pristine ZIS catalyst under dark and irradiation. (f) The structure and carrier transfer route of Ga‐ZvIS after illumination. Transient absorption spectra of (g,h) ZIS, (j,k) Ga‐ZvIS, and normalized transient absorption kinetics of (i) ZIS and (l) Ga‐ZvIS after 500 nm laser excitation.

The charge transfer between ZIS and the Ga‐ZvIS sample, induced by the introduction of Ga atoms and Zn vacancies, was investigated through in situ XPS [32]. The In 3d peak in both pristine ZIS and Ga‐ZvIS is shifted toward lower binding energy under illumination compared to the dark condition, whereas the binding energy of Zn 2p moves towards higher energy (Figure 3a,b,d,e). This demonstrates that photogenerated electrons accumulate at Ga–In domains (reduction centers), while holes localize at Zn‐vacancy regions (oxidation centers) in Ga‐ZvIS. The Ga 3d peak in Ga‐ZvIS is shifted toward lower binding energy under illumination compared to the dark condition, which suggests that photogenerated electrons will be enriched at In sites as the main reduction site due to the Ga atoms substitute In atoms through In–S layer (Figure 3c–f). This behavior reveals that Ga substitution and Zn vacancies induce asymmetric carrier redistribution, thereby enhancing charge separation and H_2_O_2_ formation.

The enhanced carrier separation kinetics and charge transportation were further elaborated by femtosecond transient absorption spectroscopy (fs‐TA spectra). According to the decay kinetics and fitted average lifetimes, the Ga‐ZvIS catalyst possesses a longer average carrier lifetime (τ_av_) compared to that of pristine ZIS (Figure 3g–l). This confirms that Ga doping and Zn vacancies enhance carrier transport dynamics and significantly suppress recombination. Specifically, the decay curve of pristine ZIS and Ga‐ZvIS catalyst can be fitted using a double‐exponential and three‐exponential model, respectively. Compared with pristine ZIS, the shortest (τ_1_) in Ga‐ZvIS corresponds to rapid electron transfer from the bulk to surface active sites, while the longest lifetime (τ_3_) reflects suppressed recombination between photogenerated electrons and trapped holes, evidencing more efficient charge separation and transport (Figure S10) [33]. The significantly extended carrier lifetime in Ga‐ZvIS reveals its superior charge transportation and reduced recombination of photogenerated carriers.

**FIGURE 4:**
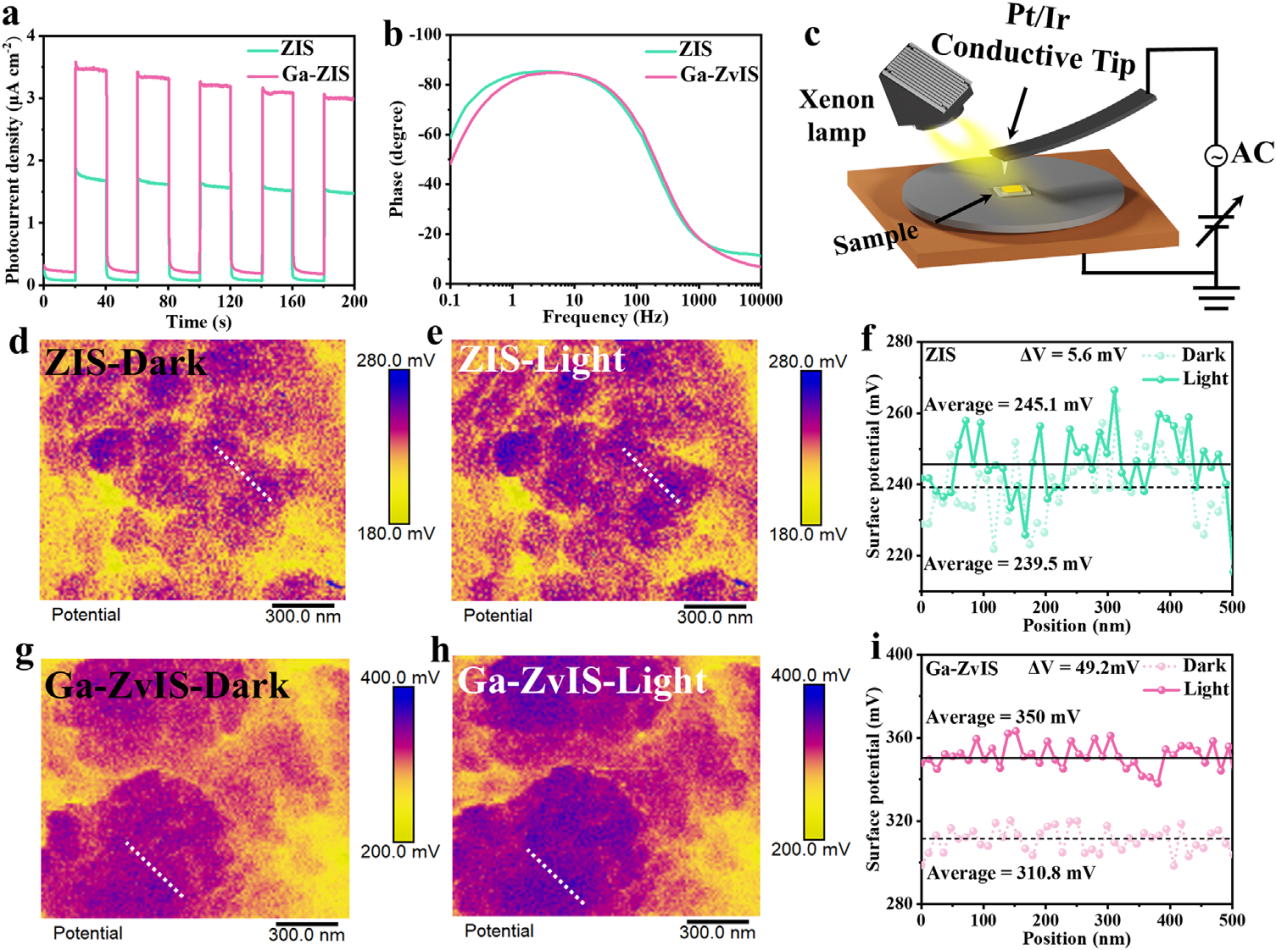
Charge separation and transport properties (a) Transient photocurrent curves and (b) Bode phase spectrum of ZIS and Ga‐ZvIS. (c) Schematic diagram of KPFM. (d–f) KPFM images and the corresponding surface potential profiles of pristine ZIS under dark and light illumination. (g–i) KPFM images and the corresponding surface potential profiles of Ga‐ZvIS under dark and light illumination.

To further confirm the enhanced carrier separation and migration, photoelectrochemical tests including transient photocurrent response and EIS test are also provided and the results show that the optimized Ga‐ZvIS catalyst exhibits a distinctly higher photocurrent density, a smaller radius of the Nyquist plot and a larger peak frequency at the minimum phase difference (Figure 4a,b; Figure S11) [34, 35]. This can be attributed to the introduction of Ga atoms and Zn vacancies in ZIS catalyst, which promote charge carrier separation and migration efficiency. Photoluminescence spectra also confirm the efficient separation and transportation of photogenerated carriers in Ga‐ZvIS catalyst (Figure S12).

Kelvin probe force microscopy (KPFM) provides an intuitive and comprehensive approach to study the separation and transfer of charge carriers [36]. The optimized Ga‐ZvIS catalyst displays the strongest potential distribution (310.8 mV) under dark conditions, superior to that of pristine ZIS (239.5 mV) (Figure 4c–i; Figures S13 and S14). This indicates the presence of a pronounced built‐in electrostatic potential in Ga‐ZvIS, which facilitates directional charge migration. Noticeably, under light illumination, the surface potentials of ZIS and Ga‐ZvIS catalyst are increased, and it reveals that the photogenerated holes are transferred to the detected surfaces of Ga‐ZvIS catalyst [37]. The surface potential shift (ΔV) of 49.2 mV for Ga‐ZvIS, compared with 5.6 mV for ZIS, further confirms the enhanced intrinsic polarization and superior charge‐separation efficiency arising from electron–hole asymmetry.

Additionally, we further investigated the light absorption properties of the photocatalysts. As‐prepared catalysts exhibit visible‐light absorption from 300 to 550 nm, and the optical band gaps of ZIS, ZvIS, Ga‐ZIS, and Ga‐ZvIS are calculated to be 2.83, 2.93, 2.83, and 2.92 eV, respectively (Figures S15 and S16). According to the equation E_vb_ = Φ + E_vb‐xps_–4.44 eV (where Φ is the work function of the XPS instrument, 4.40 eV) [19], the positions of the valence bands (VBs) of ZIS, ZvIS, Ga‐ZIS, and Ga‐ZvIS are determined to be 1.64, 1.66, 1.63, and 1.64 V (with respect to the NHE), respectively (Figure S17). The energy band structures of as‐prepared catalysts are obtained based on the above results, and all catalysts can meet the theoretical potential requirements for H_2_O_2_ production by oxygen reduction reaction (Figure S18).

### 2.3 Photocatalytic H_2_O_2_ Performance of Catalysts

**FIGURE 5:**
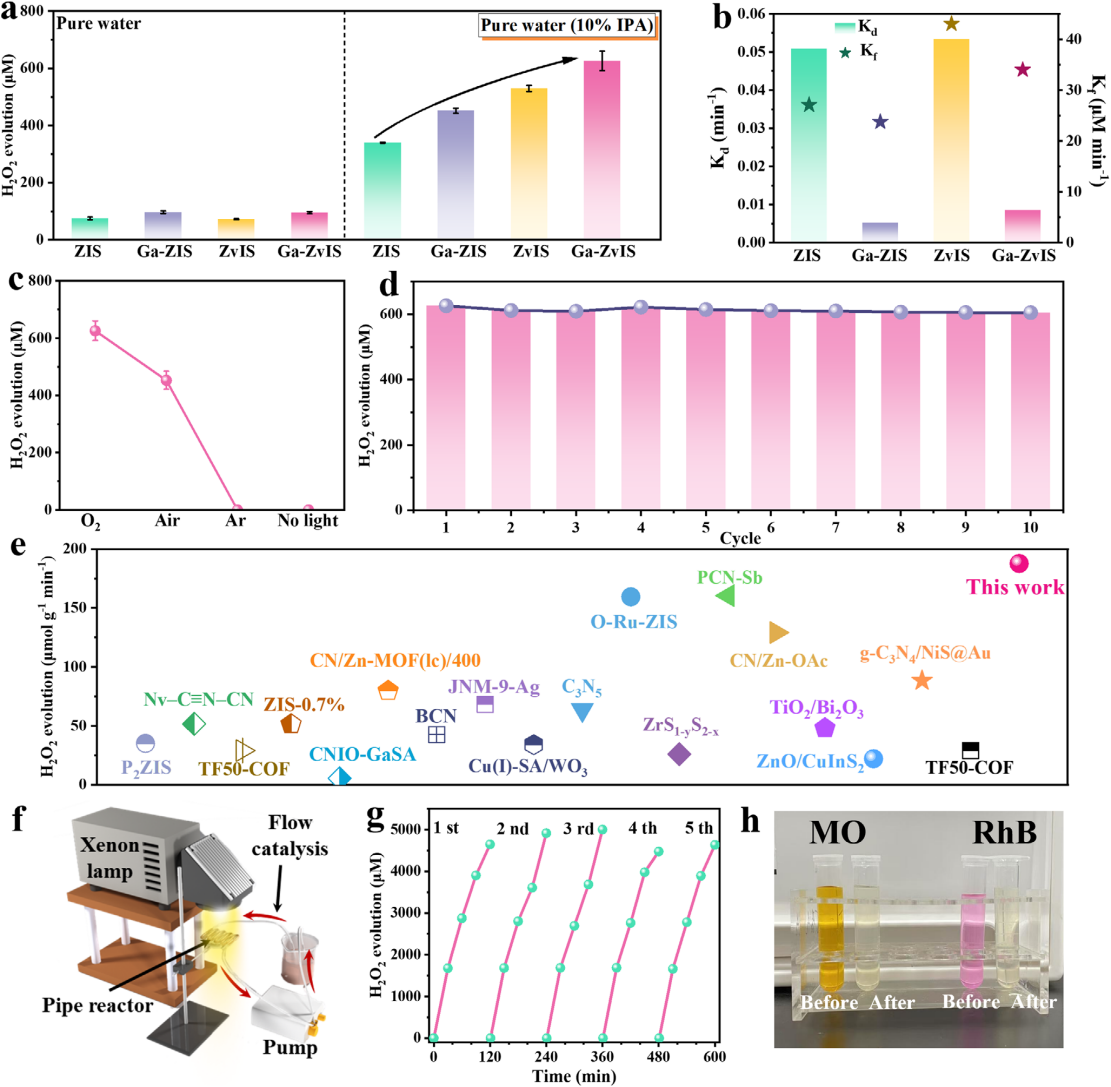
Photocatalytic H_2_O_2_ production performance (a) Photocatalytic H_2_O_2_ evolution rates of the as‐prepared photocatalysts under pure water and 10% IPA, respectively. (b) H_2_O_2_ formation rate constant (K_f_) and decomposition rate constant (K_d_) for photocatalysts. (c) Photocatalytic H_2_O_2_ evolution rate of the Ga‐ZvIS catalyst under various conditions. (d) Cycling experiments of the optimized Ga‐ZvIS catalyst. (e) Performance comparison achieved by the Ga‐ZvIS catalyst with previously reported photocatalysts. (f) Schematic diagram of a flow catalytic device for photocatalytic H_2_O_2_ production. (g) Photocatalytic H_2_O_2_ evolution rate of the Ga‐ZvIS catalyst from Figure 5f. (h) Optical photographs of MO and RhB degradation by Fenton reaction using H_2_O_2_ produced by Ga‐ZvIS from Figure 5f.

The artificial H_2_O_2_ photosynthesis performance of catalysts was first examined under an oxygen atmosphere and pure water. Compared with pristine ZIS (75.1 µm), as‐synthesized Ga‐ZIS (96.6 µm), ZvIS (72.3 µm), and Ga‐ZvIS (95.1 µm) showed similar H_2_O_2_ production performances (Figure 5a). Interestingly, under an oxygen atmosphere and 10% IPA solution conditions, the optimized Ga‐ZvIS catalyst exhibited an enhanced H_2_O_2_ production rate (625.9 µm) compared with that of pristine ZIS (340.1 µm), Ga‐ZIS (451.4 µm), and ZvIS (529.4 µm), respectively (Figure 5a, Figure S19). This suggests that Ga doping and Zn vacancies can enhance oxygen reduction and IPA oxidation performance. To achieve deeper investigations about the H_2_O_2_ production dynamics, the generation rate constant (K_f_, µm min^−1^) and decomposition rate constant (K_d_, min^−1^) of catalysts were provided through the equation reported before [38]. Interestingly, the K_d_ value of Ga‐ZIS and Ga‐ZvIS catalysts was distinctly lower than that of pristine ZIS and ZvIS, which indicates that Ga substitution weakens In**─**O bonding and reduces surface‐induced H_2_O_2_ decomposition, thereby stabilizing H_2_O_2_ formation (Figure 5b, Figure S20). Control experiments exhibited that H_2_O_2_ production was completely inhibited in the absence of light or under Ar conditions, while the H_2_O_2_ production rate can reach 625.9 and 425.7 µm under O_2_ and air condition, respectively, further suggesting the ORR route and highlighting the catalyst's optimized performance under 15°C (Figure 5c; Figure S21). Cycling experiments and several characterizations before and after the photo‐reactions confirmed that Ga‐ZvIS has superior stability and reusability (Figure 5d; Figures S22–S26). The optimized performance achieved by Ga‐ZvIS exceeded that of most previously reported organic and inorganic photocatalysts (Figure 5e, Table S2) [39, 40, 41, 42, 43, 44, 45, 46, 47, 48, 49, 50, 51, 52, 53, 54, 55, 56]. Additionally, a proof‐of‐concept continuous‐flow photoreactor demonstrated in situ Fenton‐assisted oxidation of organic contaminants using the photosynthesized H_2_O_2_, highlighting its practical applicability (Figure 5f–h; Figure S27).

### 2.4 Radical Trapping Experiments and Reaction Mechanism

**FIGURE 6:**
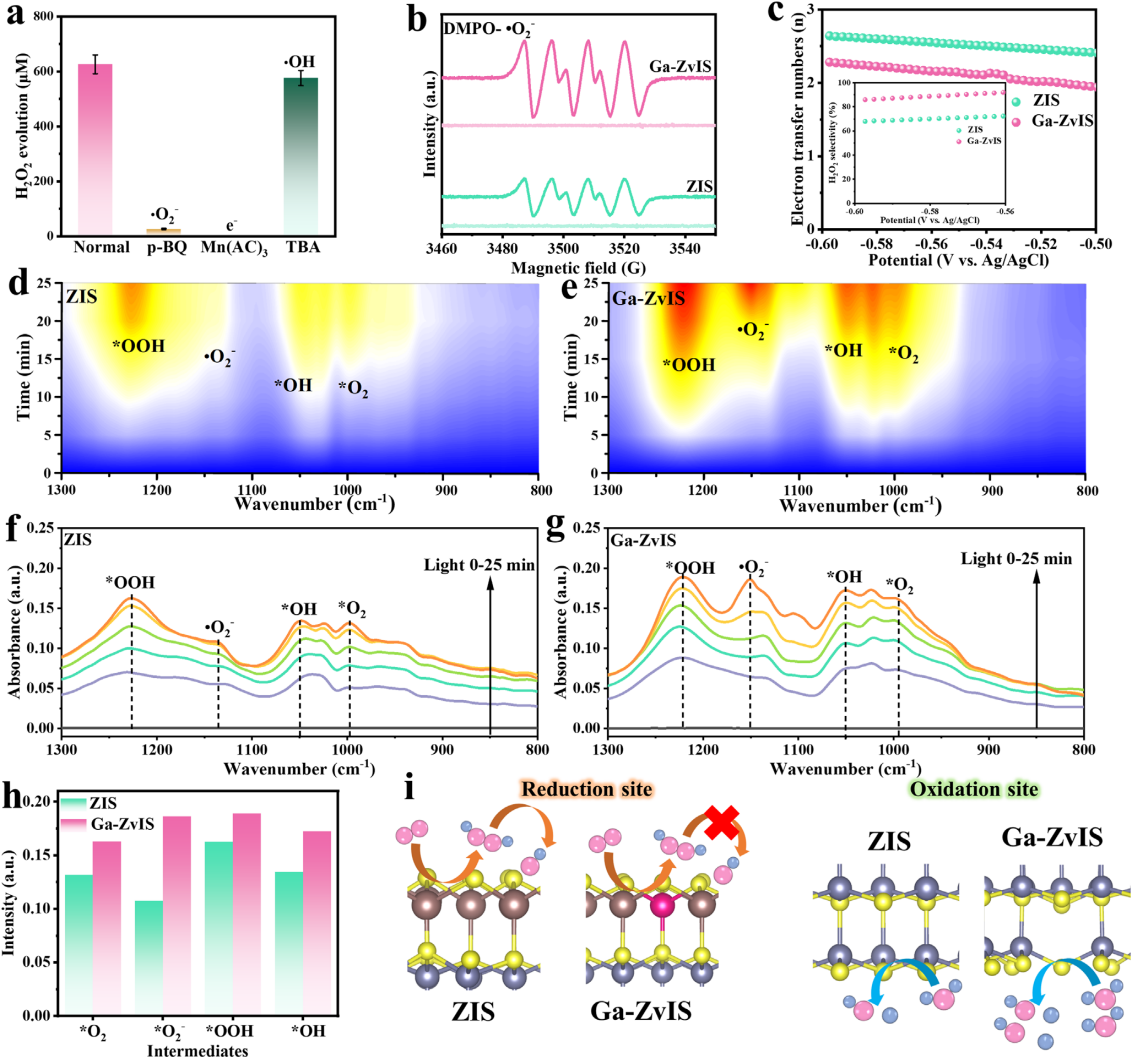
Reaction intermediates and mechanistic analysis (a) Radical trapping experiments on Ga‐ZvIS catalyst. (b) DMPO spin‐trapping ESR spectra for •O_2_^−^. (c) Electron transfer number and selectivity of H_2_O_2_ production from RRDE. In situ DRIFTS spectra recorded over (d and f) ZIS and (e and g) Ga‐ZvIS catalyst under irradiation. (h) The intermediate production of ZIS and Ga‐ZvIS with changes in the light exposure time. (i) Schematic diagram of Ga doped, Zn vacancies‐mediated ZIS for enhanced H_2_O_2_ production.

To investigate the reaction mechanism of Ga‐ZvIS catalyst, quenching experiments were provided. Introducing p‐benzoquinone (p‐BQ) and Mn(AC)_3_, a •O_2_^−^ and electron scavenger, respectively, significantly caused the decrease of H_2_O_2_ yield while no obvious reduction in H_2_O_2_ yield was detected upon the addition of tert‐butyl alcohol (TBA), a •OH scavenger (Figure 6a). This suggests the critical role of •O_2_^−^ as an intermediate in H_2_O_2_ production, with the reaction primarily driven by photogenerated electron reduction. Electron spin resonance (ESR) technique results were consistent with the radical capture experiment results that no •O_2_^−^ and •OOH signals were detected under dark conditions while the signals were observed in pristine ZIS and Ga‐ZvIS catalyst, respectively (Figure 6b, Figure S28). Meanwhile, the signals detected in Ga‐ZvIS were stronger than those in pristine ZIS, indicating that Ga‐ZvIS catalyst consistently produced intermediates that subsequently form H_2_O_2_. To investigate the electron transfer number and H_2_O_2_ selectivity, a rotating ring‐disk electrode (RRDE) was employed and the results exhibited that Ga‐ZvIS catalyst displayed an electron transfer number close to 2, higherH_2_O_2_ selectivity compared to pristine ZIS (Figure 6c, Figure S29).

In situ diffuse reflectance infrared Fourier transform spectroscopy (DRIFTS) was measured to investigate the dynamic intermediates during H_2_O_2_ evolution reaction (Figure 6d–h). The peak detected at 1200–1250 cm^−1^ was attributed to the formation of ^*^OOH intermediates while the peak observed at 1167 and 1050 cm^−1^ was ascribed to the formation of •O_2_^−^ and ^*^OH intermediates, respectively [34, 57, 58]. Under prolonged illumination and presence of oxygen and water, an increasing signal trend of •O_2_^−^ and ^*^OH intermediate in both pristine ZIS and Ga‐ZvIS catalyst was observed. Noticeably, compared with pristine ZIS, Ga‐ZvIS exhibited stronger •O_2_^−^ and ^*^OH signals, indicating that Ga substitution enhances •O_2_^−^ generation for selective H_2_O_2_ formation and stabilization, while Zn vacancies facilitate water activation and proton supply. To better understand the designed Ga‐ZvIS catalyst, based on our above results, we provide a brief schematic diagram illustrating the reaction mechanism (Figure 6i). Specifically, Ga substitution facilitates the two‐electron oxygen reduction and suppresses H_2_O_2_ decomposition, whereas Zn vacancies enhance oxidation capacity and proton mobility within ZIS.

**FIGURE 7:**
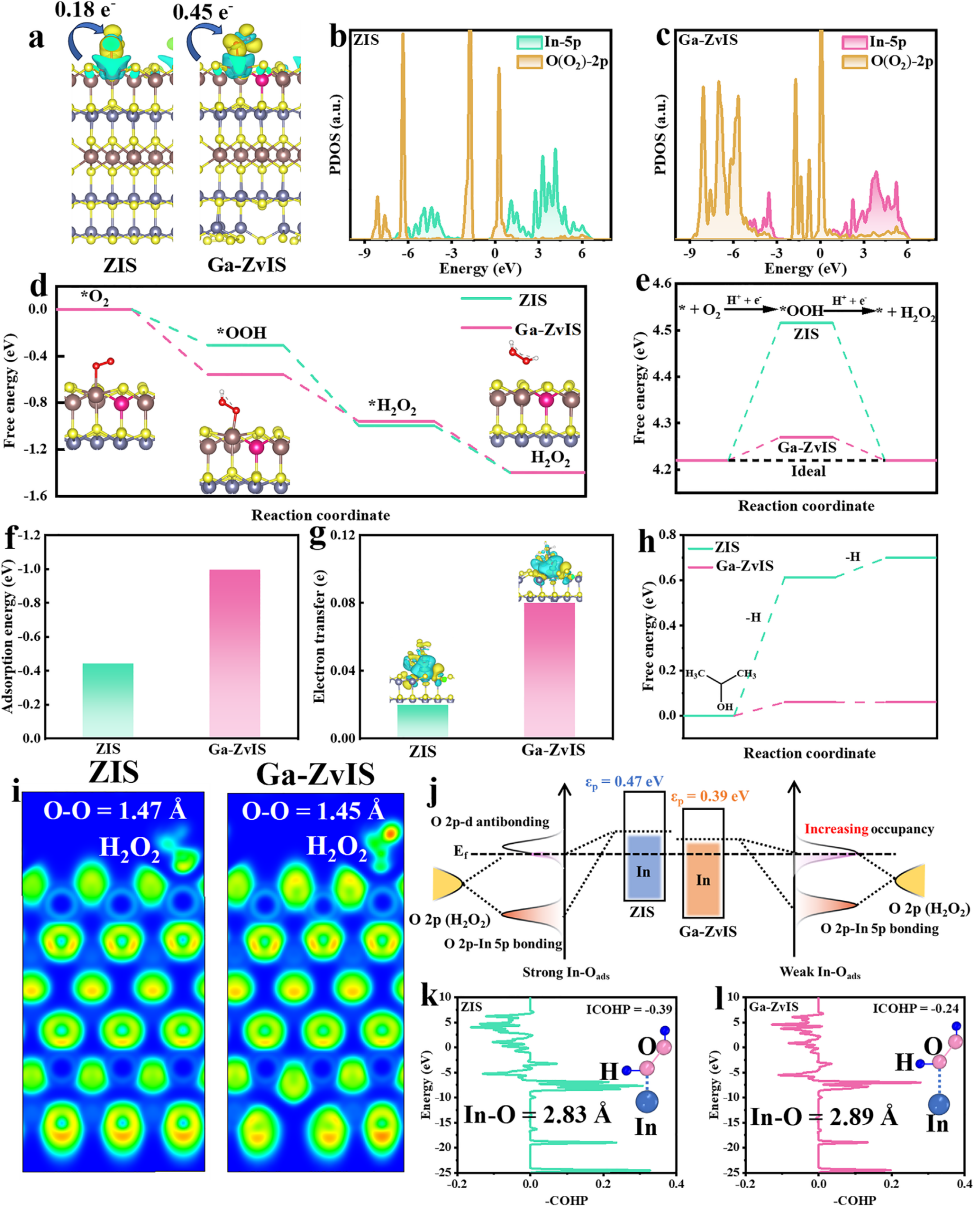
DFT analysis of reaction energetics (a) The differential charge and electron transfer between ZIS and Ga‐ZvIS in oxygen adsorption. PDOS of In 5p and O (O_2_)‐2p in (b) ZIS and (c) Ga‐ZvIS. (d) Free energy profiles of ORR and (e) ∆G^*^OOH. (f) Adsorption energy of IPA in ZIS and Ga‐ZvIS. (g) The differential charge and electron transfer between ZIS and Ga‐ZvIS in IPA adsorption. (h) Gibbs free energy of IPA dehydrogenation in ZIS and Ga‐ZvIS. (i) Electron local function (ELF) result after H_2_O_2_ adsorption on ZIS and Ga‐ZvIS. (j) Diagram illustrating the reduced p‐band center of indium to weaken In─O bonds. (k and l) COHP data of In─O bonds in ZIS and Ga‐ZvIS.

To elucidate the synergistic effects of Ga‐on‐In substitution and Zn vacancies on the formation of H_2_O_2_, comprehensive density functional theory (DFT) analyses were performed. Differential charges between the O_2_ and ZIS, Ga‐ZvIS were analyzed and the results exhibited that the strongest interactions existed between Ga‐ZvIS and O_2_ (Figure 7a). This reveals that Ga substitution and Zn vacancies collectively induce asymmetric charge redistribution and enhance electron transfer from ZIS to O_2_, facilitating H_2_O_2_ formation. PDOS results showed that hybridization of O 2p orbitals and In 5p orbitals in Ga‐ZvIS catalyst was much stronger than that of pristine ZIS, confirming that introducing Ga element and Zn vacancy greatly facilitated the interaction between the O_2_ molecule and the catalyst surface (Figure 7b,c). The Gibbs free energies of H_2_O_2_ evolution by ORR reaction and the free energies of ^*^OOH intermediates (∆G_*OOH_) of pristine ZIS and Ga‐ZvIS samples are further presented and the ∆G_*OOH_ values are 4.52 and 4.27 eV, respectively (Figure 7d,e). Figure 7f,g displayed the adsorption energy and charge transfer number of IPA toward catalysts and the results showed that Ga‐ZvIS exhibited the optimal IPA activation capacity. Meanwhile, the Gibbs free energies of IPA dehydrogenation by oxidation reaction towards different catalysts were provided as well (Figure 7h). The results demonstrated that Ga‐ZvIS catalyst had a significantly lowered energy barrier (0.06 eV) than that of pristine ZIS (0.61 eV), suggesting that Zn vacancy can be beneficial for promoting the IPA dehydrogenation reaction and providing sufficient protons to be involved in the H_2_O_2_ production process. Furthermore, the electron localization function analysis displayed that the gradual shortening of the O─O bond length in H_2_O_2_ was observed in the optimized Ga‐ZvIS catalyst (Figure 7i). Ga‐on‐In substitution downshifts the In 5p‐band center, weakening the In─O bond interaction and stabilizing H_2_O_2_, while Zn vacancies primarily localize holes and facilitate proton generation (Figure 7j; Figures S30–S32). Specifically, according to the ICOHP results, it can be noted that the interaction between ZIS and H_2_O_2_ in Ga‐ZvIS catalyst can be effectively reduced, which can stabilize the O─O bond and inhibit the decomposition of H_2_O_2_ (Figure 7k,l). These combined features are highly beneficial for enhancing the photocatalytic H_2_O_2_ production under ambient conditions.

## 3 Conclusions

In summary, the integration of Ga‐on‐In substitution and Zn vacancies in Zn_3_In_2_S_6_ (Ga‐ZvIS) enables efficient charge separation, sufficient proton supply, and improved H_2_O_2_ stabilization, resulting in markedly enhanced photocatalytic H_2_O_2_ production. The optimized Ga‐ZvIS catalyst achieved an outstanding H_2_O_2_ generation rate of 187.8 µmol g^−^^1^ min^−^^1^, surpassing most reported systems. The improved activity originates from electron–hole asymmetry and the resulting built‐in electrostatic potential that promotes directional carrier transport. Ga‐on‐In sites weaken the In─O bond to suppress H_2_O_2_ decomposition, while Zn‐vacancy domains facilitate hole extraction and proton generation for the two‐electron oxygen reduction reaction. Together, these findings establish a clear mechanistic basis for coupling electron–hole asymmetric charge regulation with surface bond modulation, thereby defining a general design framework for photocatalytic H_2_O_2_ photosynthesis. While the present study is demonstrated using Zn_3_In_2_S_6_ as a model system, the underlying principle of spatially separating reduction and oxidation domains through cooperative defect engineering is expected to be broadly applicable, and future studies may further explore the extension of this strategy to other material platforms and reaction conditions.

## Conflicts of Interest

The authors declare no conflicts of interest.

## Supporting information




**Supporting File**: adma72735‐sup‐0001‐SuppMat.docx.

## Data Availability

The data that support the findings of this study are available from the corresponding author upon reasonable request.
