# Supplementary material for: Ga‐on‐In Substitution with Zn Vacancies in Zn3In2S6 Induces Electron–Hole Asymmetry and In─O Bond Weakening for Coupled Two‐Electron Oxygen Reduction and H2O2 Stabilization
Source: Adv Mater. 2026 Mar 19;38(23):e22831. doi: 10.1002/adma.202522831 (PMC13103633; doi:10.1002/adma.202522831)
Supplement: Supplementary file 1 — Supporting File: adma72735‐sup‐0001‐SuppMat.docx. [file ADMA-38-e22831-s001.docx]

**Ga-on-In Substitution with Zn Vacancies in Zn_3_In_2_S_6_ Induces Electron–Hole Asymmetry and In–O Bond Weakening for** **Coupled Two-Electron Oxygen Reduction and H_2_O_2_ Stabilization**

Xiaowen Ruan,^#[1]^ Chunsheng Ding,^#[1]^ Dongxu Jiao,*^[2]^ Jing Leng,^[3]^ Minghua Xu,^[4]^ Bonan Li,^[1]^ Zhipeng Yu,^[5]^ Xiaoqiang Cui,^[4]^ Jimmy C. Yu,^[6]^ Yongfa Zhu,*^[7]^ and Sai Kishore Ravi*^[1]^

^[1]^ School of Energy and Environment, City University of Hong Kong, Tat Chee Avenue, Kowloon, Hong Kong SAR, 999077, China

^[2]^ College of Chemistry, Chemical Engineering and Resource Utilization, Northeast Forestry University, Harbin 150040, China

^[3]^ State Key Laboratory of Chemical Reaction Dynamics, Dalian Institute of Chemical Physics, Chinese Academy of Sciences, Dalian 116023, China

^[4]^ School of Materials Science and Engineering, Key Laboratory of Automobile Materials of MOE, Jilin University, Changchun 130012, China

^[5]^ International Iberian Nanotechnology Laboratory (INL), Avenida Mestre Jose Veiga, 4715-330 Braga, Portugal

^[6]^ Department of Chemistry, Chinese University of Hong Kong, Shatin, New Territories 999077, Hong Kong, China

^[7]^ Department of Chemistry, Tsinghua University, Beijing 100084, China

#These authors contributed equally

*Corresponding Author: Sai Kishore Ravi; Dongxu Jiao; Yongfa Zhu

E-mail: skravi@cityu.edu.hk; dxjiao@nefu.edu.cn; zhuyf@tsinghua.edu.cn

**Table of Contents**

[**1.** **Chemicals and materials** 3](#_Toc211327857)

[**2.** **Synthesis of samples** 3](#_Toc211327858)

[**2.1 The synthesis of** **Gallium doped Zn_3_In_2_S_6_ (Ga-ZIS)** 3](#_Toc211327859)

[**2.2 The synthesis of** **Gallium doped Zn vacancy Zn_3_In_2_S_6_ (Ga-ZvIS)** 3](#_Toc211327860)

[**3.** **Characterization** 4](#_Toc211327861)

[**4.** **Photocatalytic H_2_O_2_ production** 4](#_Toc211327862)

[**5.** **Photoelectrochemical measurements** 5](#_Toc211327863)

[**6.** **The rotating ring disk electrode (RRDE) measurement** 5](#_Toc211327864)

[**7.** **In situ Fourier transform infrared (FTIR) Spectroscopy** 6](#_Toc211327865)

[**8.** **Computational methods** 6](#_Toc211327866)

[**9.** **Figure S1-S32** 8](#_Toc211327867)

[**10.** **Table. S1-S2** 24](#_Toc211327868)

[**11. References** 26](#_Toc211327869)

1. **Chemicals and materials**

Zinc sulphate heptahydrate (ZnSO_4_.7H_2_O, 99.995%), potassium hydrogen phthalate (99.8%), para-benzoquinone (p-BQ, 99%), and absolute ethanol were purchased from Macklin Biochemical Technology Co., Ltd. Thioacetamide (TTA, 98%), phosphate buffer (pH=7.0), indium trichloride tetrahydrate (InCl_3_.4H_2_O, 99.99%), tert-butyl alcohol (TBA, 99.5%), Manganese acetate dihydrate (MnAC_3_, 97%), Gallium nitrate hydrate (Ga(NO_3_)_3_·xH_2_O, 99.9%), Ethylene glycol (EG, 99.9%), potassium iodide (KI, 99%), Isopropyl Alcohol (IPA, 99.7%) were purchased from Aladdin Biochemical Technology Co., Ltd. All the purchased drugs can be used directly without any purification.

1. **Synthesis of samples**

**2.1 The synthesis of** **Gallium doped Zn_3_In_2_S_6_ (Ga-ZIS)**

3 mmol ZnSO_4_.7H_2_O and 2 mmol InCl_3_.4H_2_O were dissolved in 30 ml of deionized water, after stirring for 30 min, add 88.6 μmol Ga(NO_3_)_3_·xH_2_O to the above solution and then continue stirring for 15 min. Finally, added 12 mmol TAA and stirring for 30 min, the mixture was transferred to a 50 ml Teflon-lined stainless-steel reactor and the reaction time and temperature were set at 720 min and 160 °C, respectively. The precipitate was collected by centrifugation and washed three times with distilled water and ethanol, then dried in a vacuum oven at 60 °C. The preparation of Zn_3_In_2_S_6_ (ZIS) is similar to that of Ga-ZIS, except that Ga(NO_3_)_3_·xH_2_O is not added.

**2.2 The synthesis of** **Gallium doped Zn vacancy Zn_3_In_2_S_6_ (Ga-ZvIS)**

For Ga-ZvIS, the synthesis method is similar to that of Ga-ZIS, except that 30 mL H_2_O is replaced with 10 mL H_2_O/20 mL EG. In addition, for ZvIS, the synthesis method is similar to that of Ga-ZvIS, except that Ga(NO_3_)_3_·xH_2_O is not added.

1. **Characterization**

The crystal structure is revealed by the powder X-ray diffraction (XRD) on a Bragg-Brentano diffractometer (D8-tools, Germany) equipped with a Cu Kα source, and the scanning region was from 10° to 80°. The morphology of the samples was from a field emission scanning electron microscope (FESEM) (Hitachi, SU8010, Japan). The transmission electron microscopy (TEM) and high-resolution TEM (HRTEM) images were acquired by a JEOLJEM-2100F (UHR) Field Emission Transmission Electron Microscope. X-ray photoelectron spectroscopy (XPS) was performed on a Thermo ESCALAB 250Xi instrument with Al KαX-ray radiation. In-situ XPS was conducted under the same condition, except that ultraviolet light irradiation. The UV-visible absorption spectra of the samples were obtained by UV-vis spectrophotometer (Shimadzu, UV-2550, and Japan). With the excitation wavelength of 365 nm, SHIMADZU RF-6000 was used to analyze the photoluminescence (PL) spectra of the photocatalysts. Electron spin resonance (ESR) analysis was performed using electron spin resonance spectrometer (Jeol/JES-FA200). The femtosecond transient absorption setup is based on a regenerative amplified Ti:sapphire laser system from Coherent (800 nm, 35 fs, 6 mJ pulse^-1^, and 1 kHz repetition rate), nonlinear frequency mixing techniques and the Femto-TA100 spectrometer (Time-Tech Spectra LLC). Fourier-transform infrared (FTIR) spectra were conducted on a Bruker INVENIO R infrared spectrophotometer. The surface potential of the samples was tested by KPFM (Bruker, Dimension Icon).

1. **Photocatalytic H_2_O_2_ production**

Photocatalytic H_2_O_2_ production was carried out under sacrificial agents and O_2_ conditions. Specifically, 5 mg of photocatalyst was dispersed in 30 mL ultrapure water (10 % IPA), immediately followed by switching on the Xenon lamp (350-780 nm) for the photocatalytic reaction (Unless otherwise specified, the reaction time is 20 minutes). The generation of H_2_O_2_ was investigated by iodometry, specifically, 1 mL of the solution removed from the reactor was added to 1 mL of 0.4 M aqueous potassium iodide (KI) and 1 mL of 0.1 M aqueous potassium hydrogen phthalate, the solution was then analyzed using a UV-visible spectrophotometer at 350 nm.

Decomposition experiments were performed under argon on commercial H_2_O_2_ for degradation. Specifically, 5 mg of catalyst was placed in 30 ml of commercial H₂O₂ solution with a concentration of 1 mM, followed by the introduction of argon gas into the solution for 20 minutes. Finally, the light was turned on for irradiation for 20 minutes. The changes in the concentration of H_2_O_2_ solution before and after irradiation were compared. The concentration of H_2_O_2_ before the reaction was C_0_, while the concentration after the reaction was C. The decomposition rate constant (K_d_, min^-1^) of H_2_O_2_ follows first-order kinetics and can be calculated using the following formula: K_d_ = -ln(C/C_0_)/t, as for the H_2_O_2_ theoretical generation rate constant (K_f_, μM min^-1^), using the following formula:

$$K_{f}=\frac{K_{d}C_{{H_{2}O}_{2}}}{(1-e^{{-k}_{d}t})}$$

Among them, $C_{{H_{2}O}_{2}}$is the concentration of H_2_O_2_ produced by the photocatalyst.

1. **Photoelectrochemical measurements**

Photoelectrochemical experiments were performed on CHI650D electrochemical workstation with a conventional three electrode cell using Pt as the counter electrode, an Ag/AgCl electrode as the reference electrode. The preparation of the working electrode is as follows: 5 mg of photocatalyst was dispersed in mixed solution (2 mL) of water, isopropyl alcohol and Nafion solution (1:1:0.025) to obtain a slurry by ultrasonication 30 min. Apply the catalyst to the FTO using a spray gun and control the area to 1 cm^2^, and 0.5 M Na_2_SO_4_ as electrolyte. The light was provided by a 300 W Xe lamp.

1. **The rotating ring disk electrode (RRDE) measurement**

RRDE test was used to evaluate the number of transferred electrons (n) and H_2_O_2_ selectivity in the ORR reaction. The RRDE tests were conducted in an O_2_-saturated phosphate buffer (pH=7.0) solution with a rotating speed of 1600 rpm. The number of transferred electrons (n) is calculated according to the following formula:

$$n=4\times\frac{I_{d}}{I_{d}+I_{r}/N}$$

The selectivity of H_2_O_2_ is calculated by the following formula:

$$H_{2}O_{2}\%=2\times\frac{I_{r}/N}{I_{d}+I_{r}/N}\times100\%$$

where I_r_ is the ring current, I_d_ is the disk current, and N is the collection efficiency (N = 0.37).

1. **In situ Fourier transform infrared (FTIR) Spectroscopy**

The samples were pressed and placed into a reaction chamber and high purity helium gas was introduced for 60 min to remove small molecules such as H_2_O, O_2_ and CO_2_ adsorbed on the surface of the specimen. The samples were immediately scanned using IR spectroscopy and the results were used as a background baseline. Then 30 mL∙min^-1^ of O_2_ (containing water vapor 5%) was injected into the reaction chamber. Under dark conditions, the samples were subjected to an adsorption reaction for 60 min, and the adsorption state on the surface was examined every 5 min. Subsequently, the inlet and outlet of the reaction chamber were closed, and the photocatalytic reaction was carried out under full-spectrum irradiation for 25 min, and the infrared spectra were recorded at given time intervals, with the infrared spectral scanning area in the range of 4000-800 cm^-1^.

1. **Computational methods**

All spin−polarized density functional theory (DFT) calculations were carried out employing a plane−wave basis set as implemented in the Vienna Ab Initio Simulation Package (VASP)^1^. The electrons-ion interactions were described using the projector augmented wave (PAW) potential.^2, 3^ The exchange–correlation interactions described using the Perdew–Burke–Ernzerhof (PBE) functional within the generalized gradient approximation (GGA),^4^ with the plane wave energy cutoff of 500 eV. The convergence criterion for the residual force and energy was set to 0.05 eV Å^−1^ and 10^−5^ eV, respectively. The DFT-D3 empirical dispersion correction scheme was applied to account for the van der Waals interactions.^5^ A 2 × 2 × 1 k-point grid was used for geometric optimization. The change in the Gibbs free energy change (Δ*G*) for each possible step during the reaction process was obtained using the computational hydrogen electrode (CHE) model,^6, 7^ expressed as follows: Δ*G* = Δ*E* + Δ*E_ZPE_* − *T*Δ*S*, where the reaction energy (Δ*E*) can be directly obtained by analyzing the DFT total energies. The zero-point energy difference (Δ*E*_ZPE_) between the products and the reactants can be computed from the vibrational frequencies. Δ*S* is the change in entropy between the products and the reactants at room temperature (T = 298.15 K).

1. **Figure S1-S32**


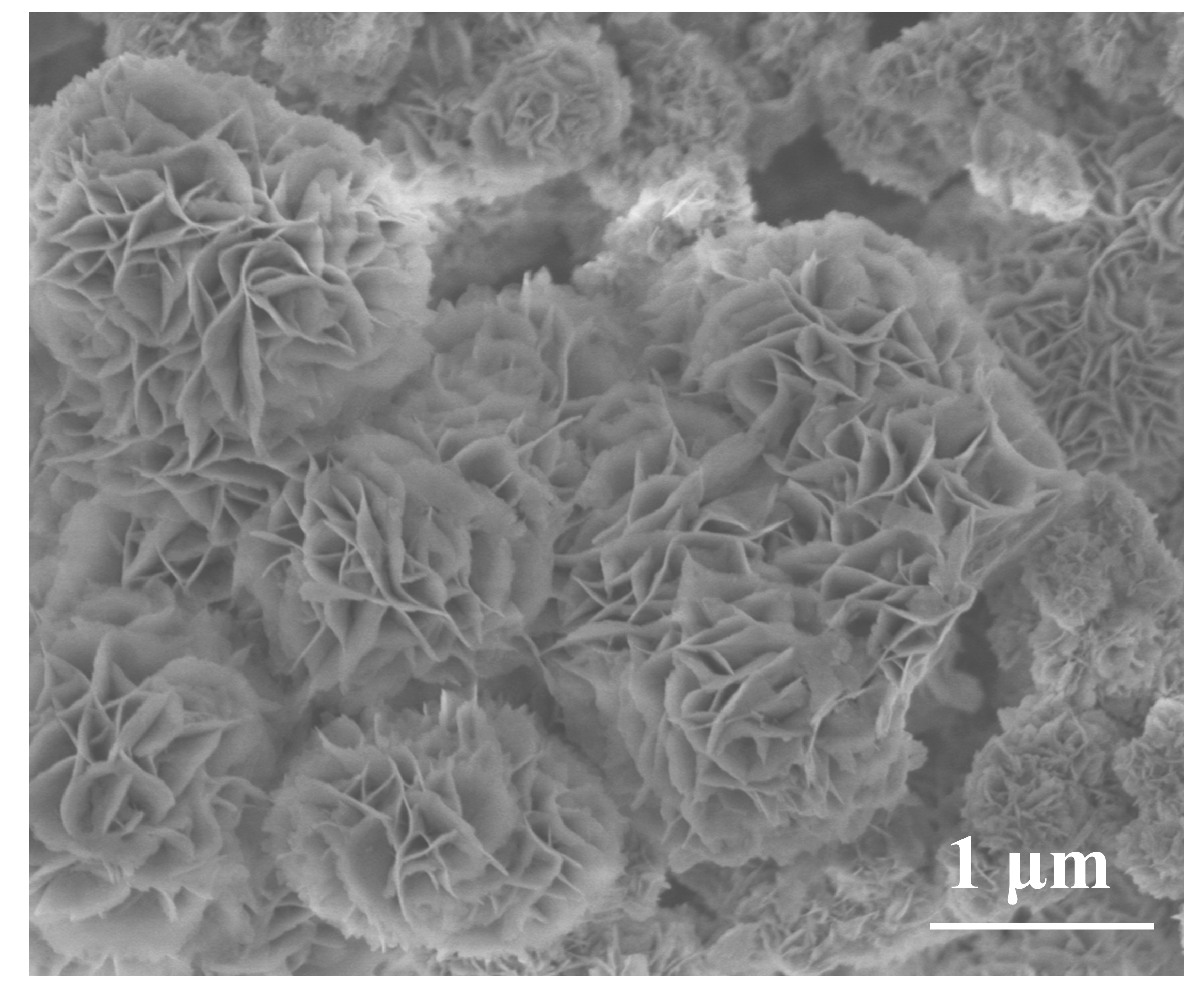


**Figure S1** SEM image of Ga-ZIS.


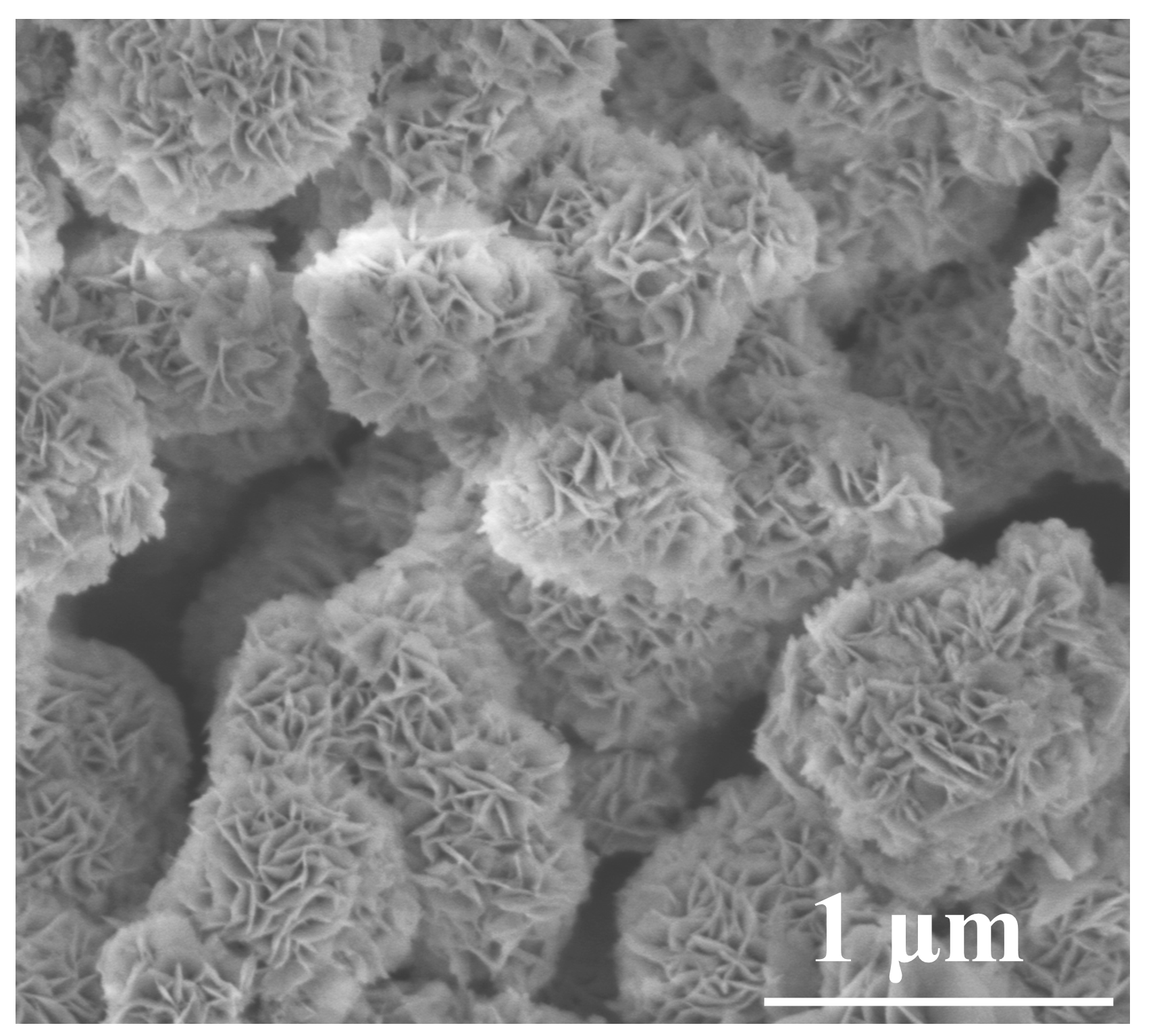


**Figure S2** SEM image of ZvIS.


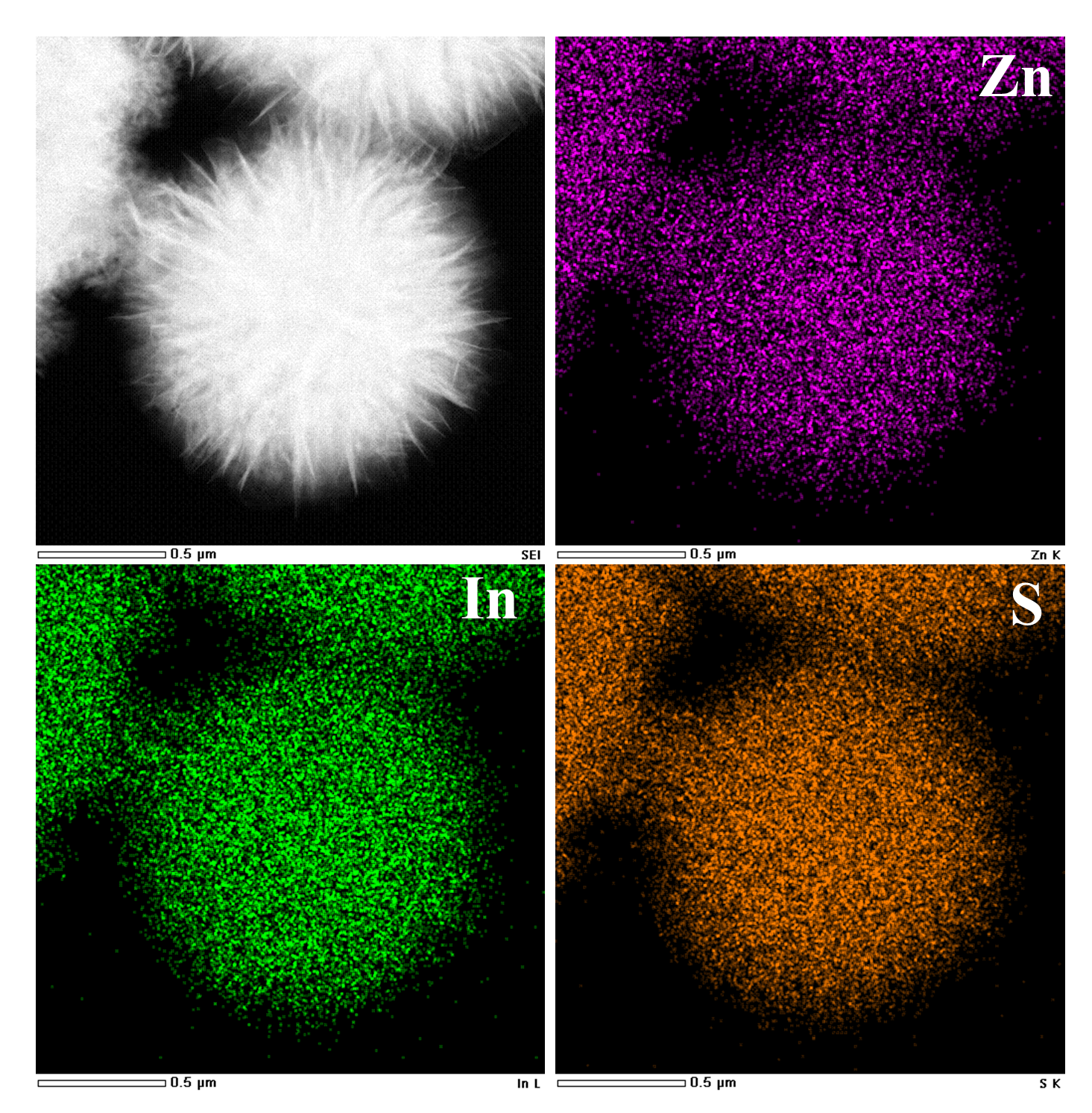


**Figure S3** Element distribution mapping images of ZIS


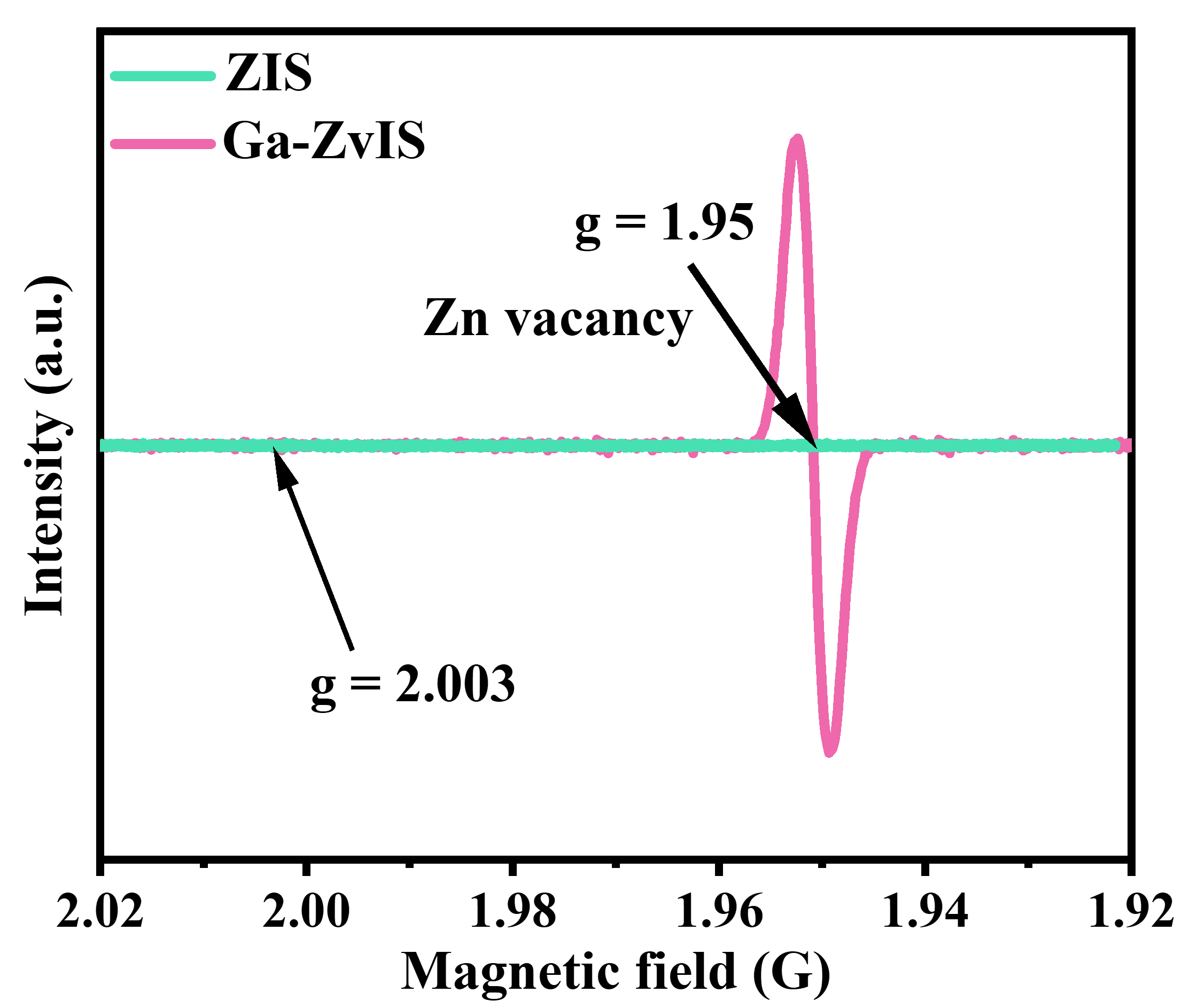


**Figure S4** ESR of ZIS and Ga-ZvIS


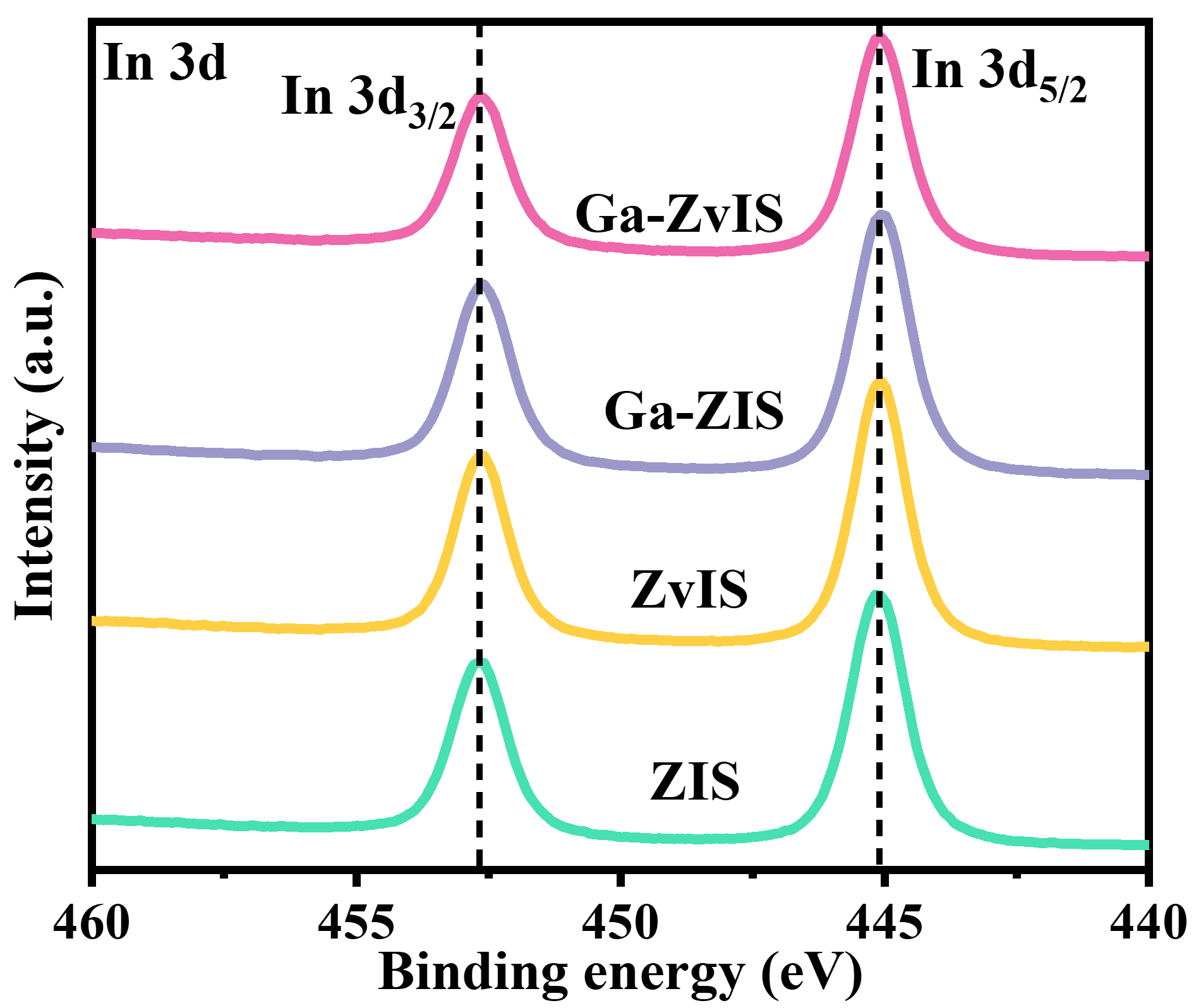


**Figure S5** In 3d XPS spectra of samples


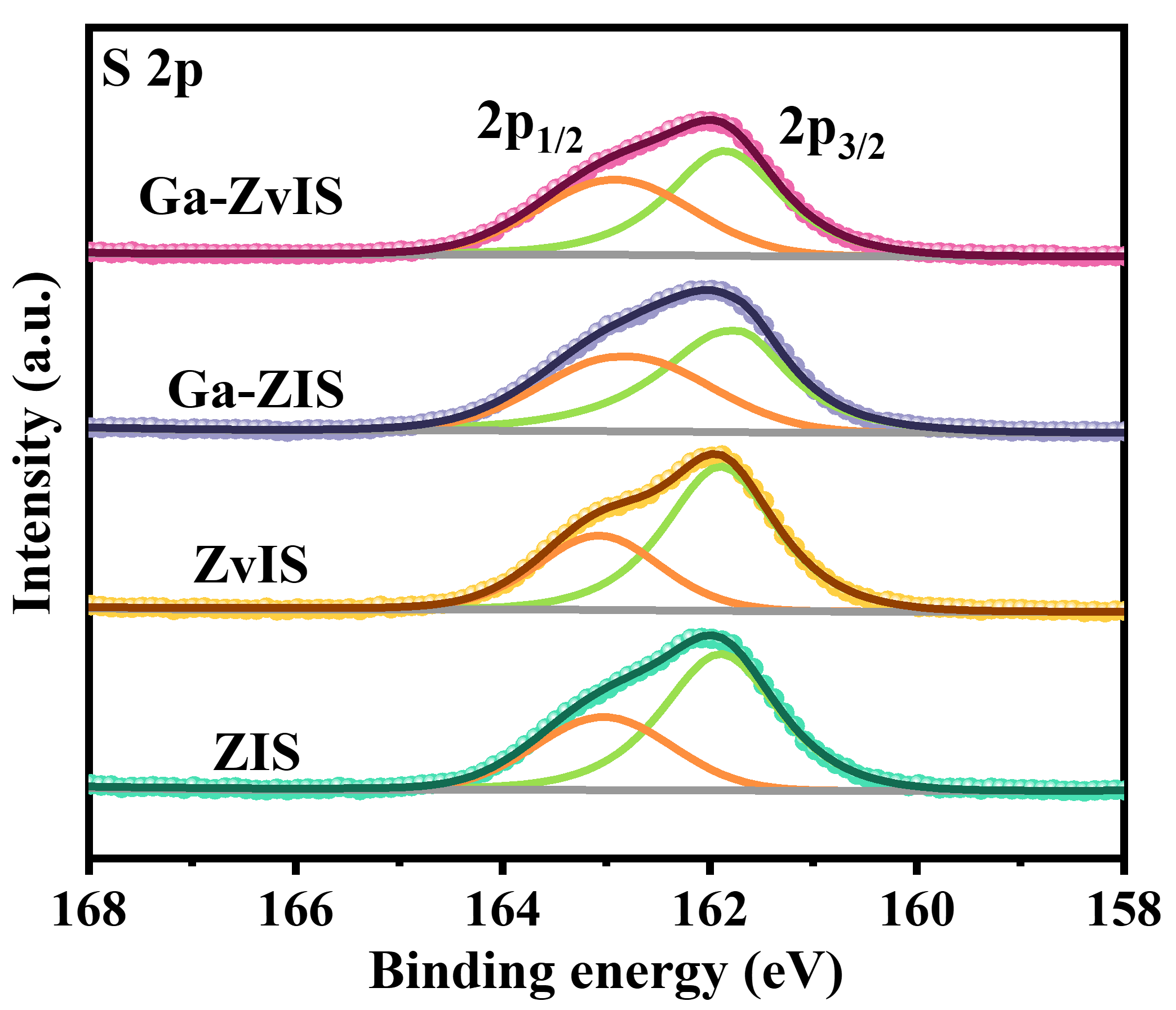


**Figure S6** S 2p XPS spectra of samples


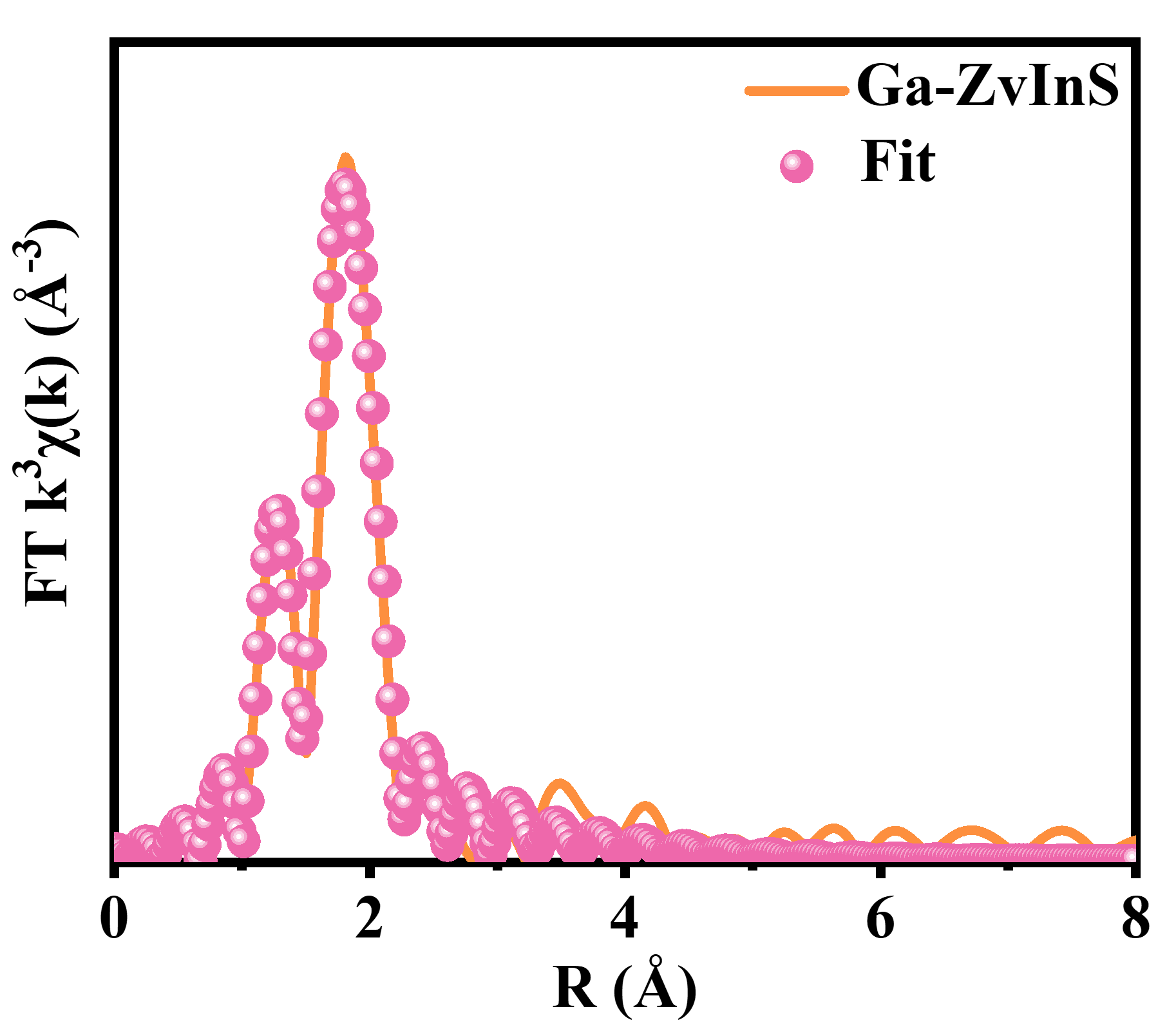


**Figure S7** Ga K-edge EXAFS fitting curves of Ga-ZvIS at R space.


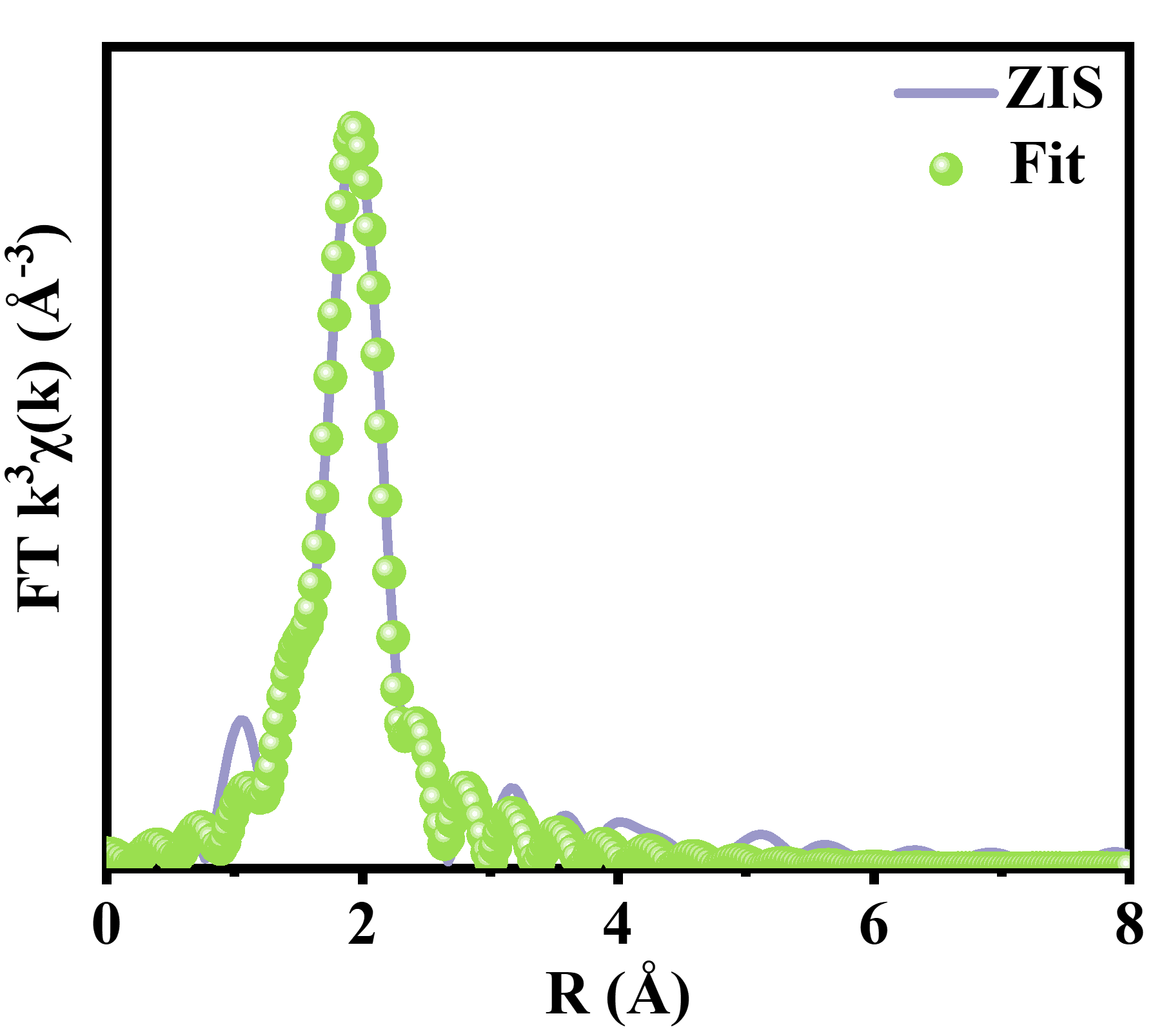


**Figure S8** In K-edge EXAFS fitting curves of ZIS at R space.


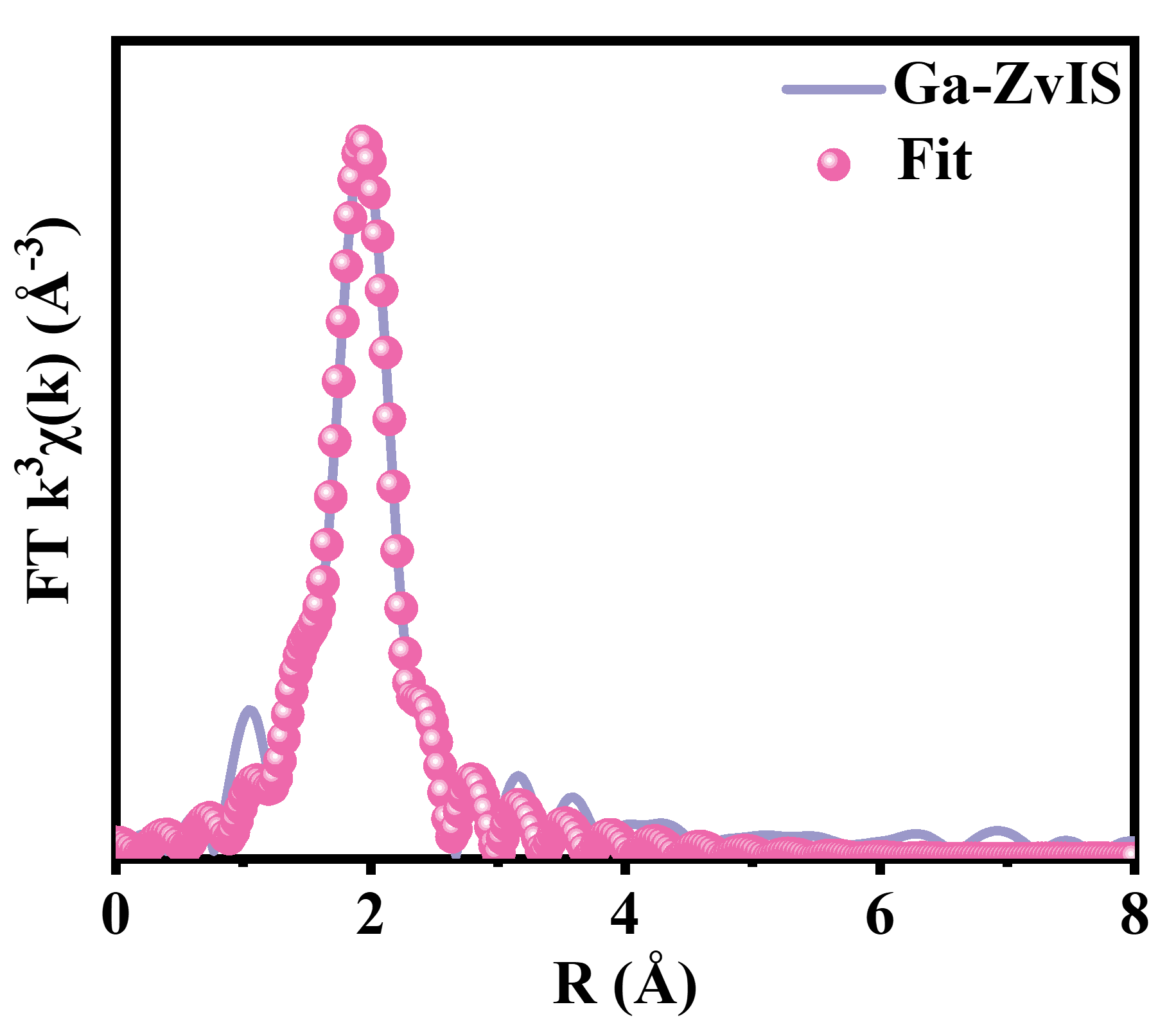


**Figure S9** In K-edge EXAFS fitting curves of Ga-ZvIS at R space


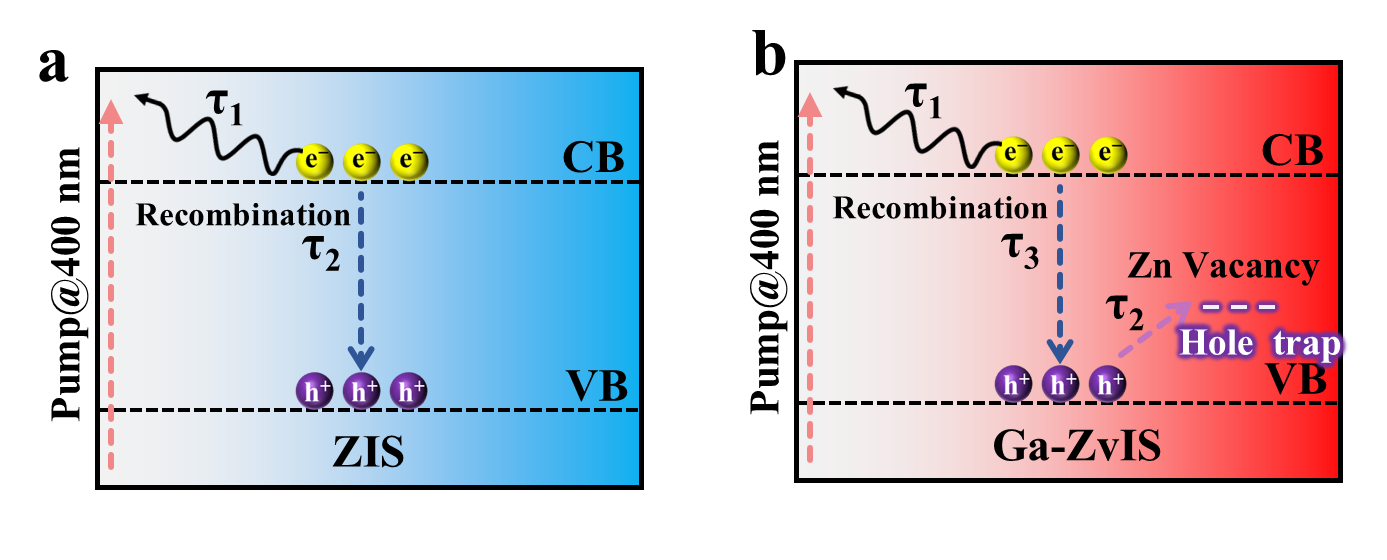


**Figure S10** Schematic Diagram of Photogenerated Carrier Transport in (a) ZIS and (b) Ga-ZvIS


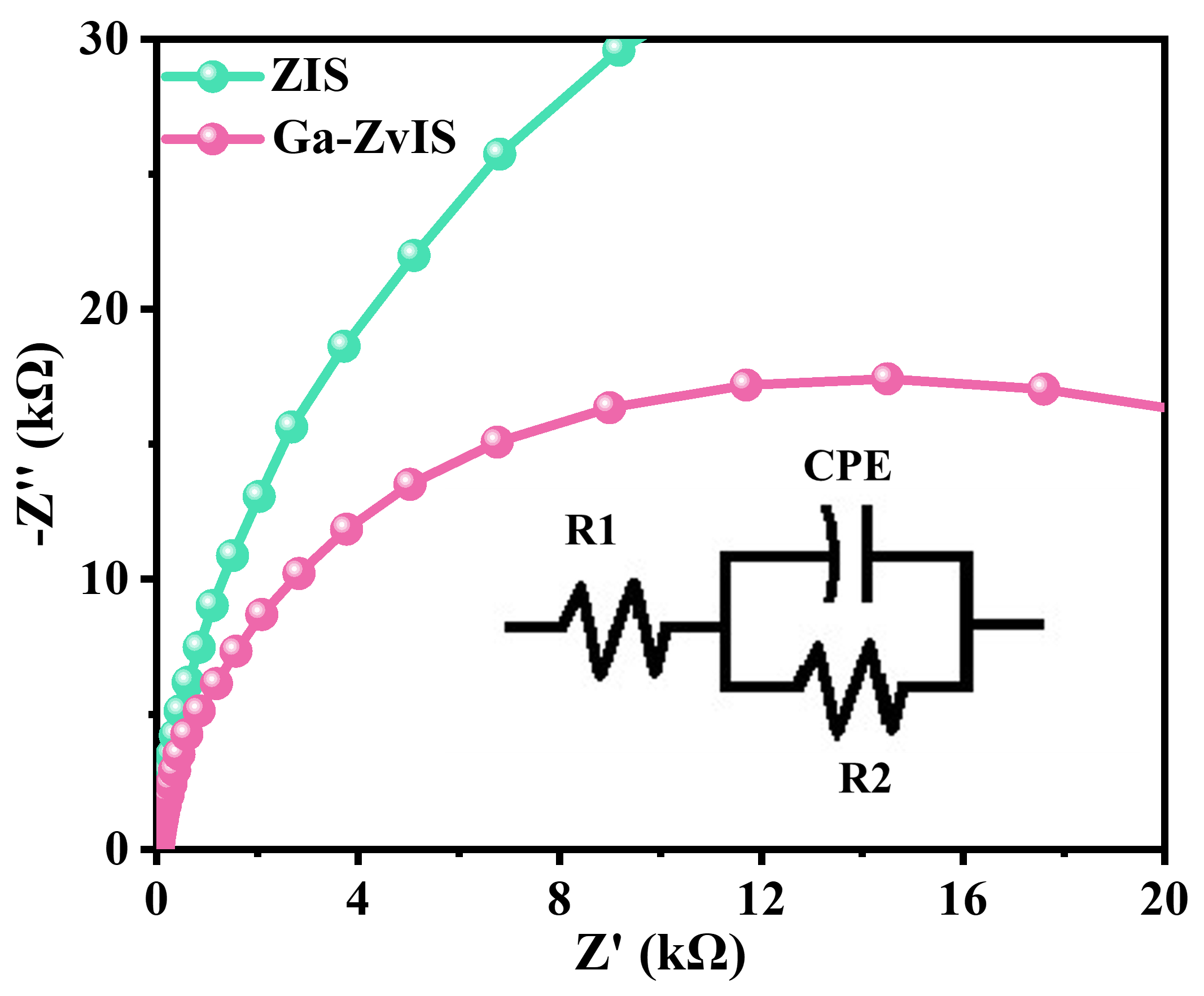


**Figure S11** EIS Nyquist plots of the samples


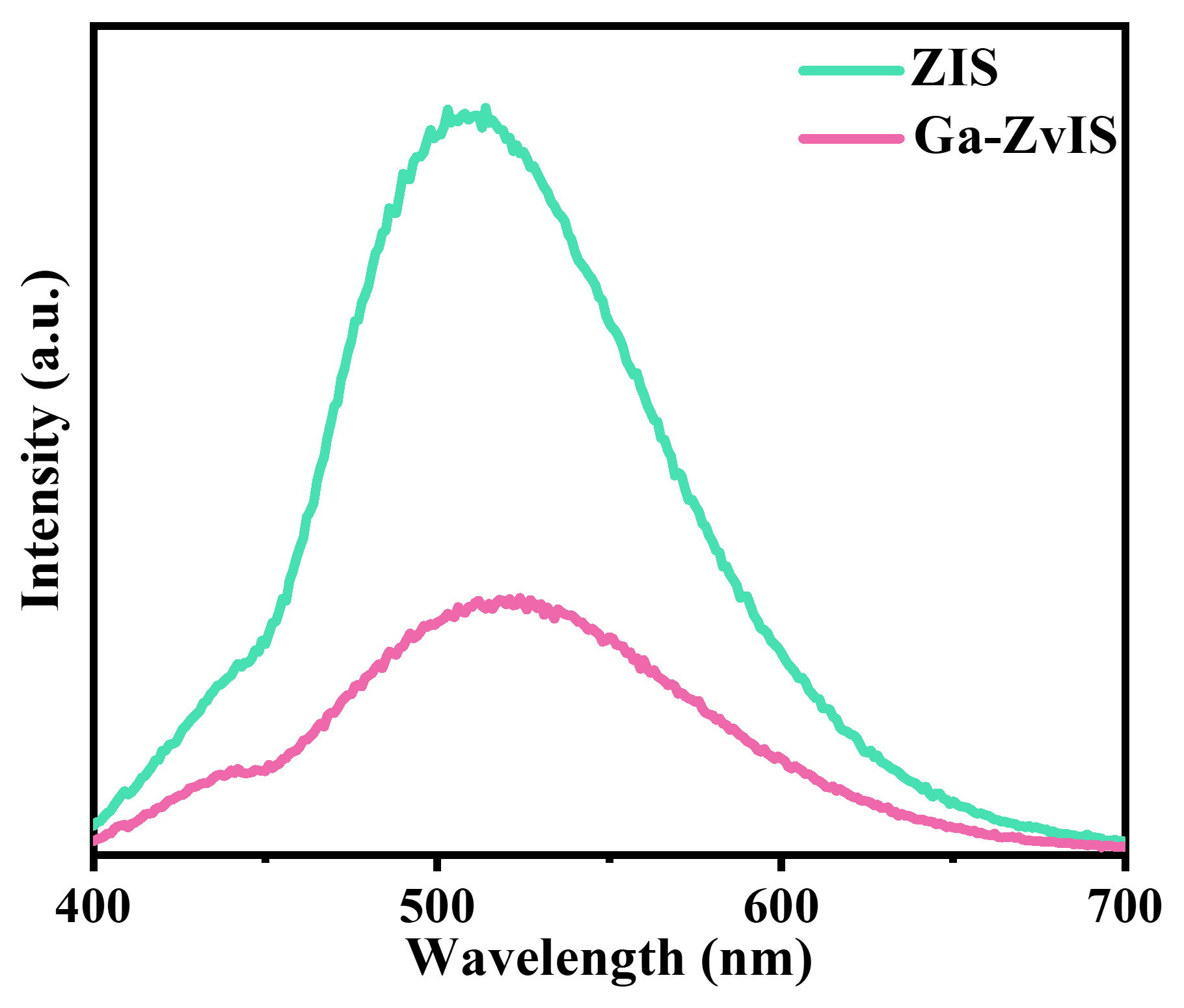


**Figure S12** Photoluminescence spectrum of the samples


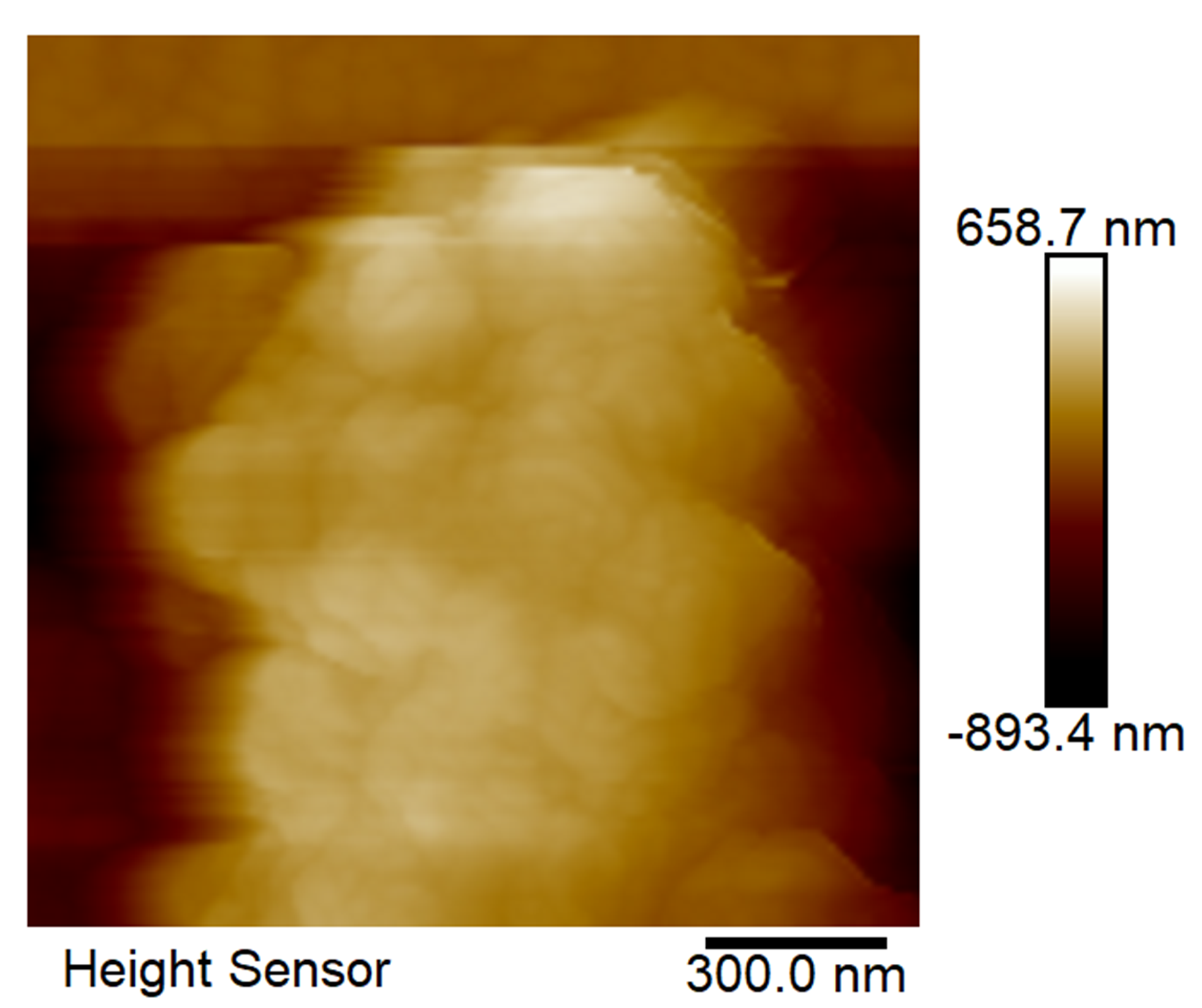


**Figure S13** AFM image of ZIS


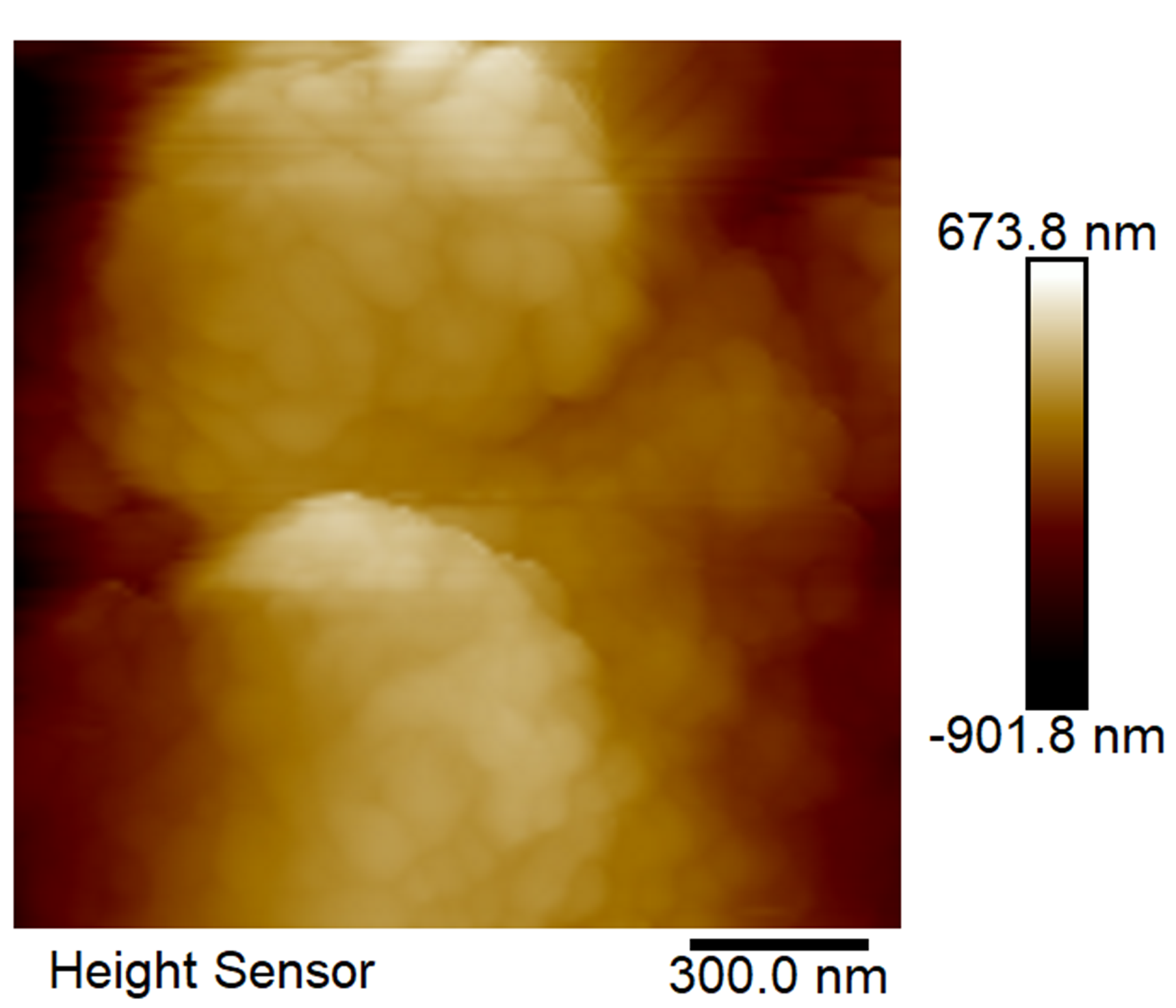


**Figure S14** AFM image of Ga-ZvIS


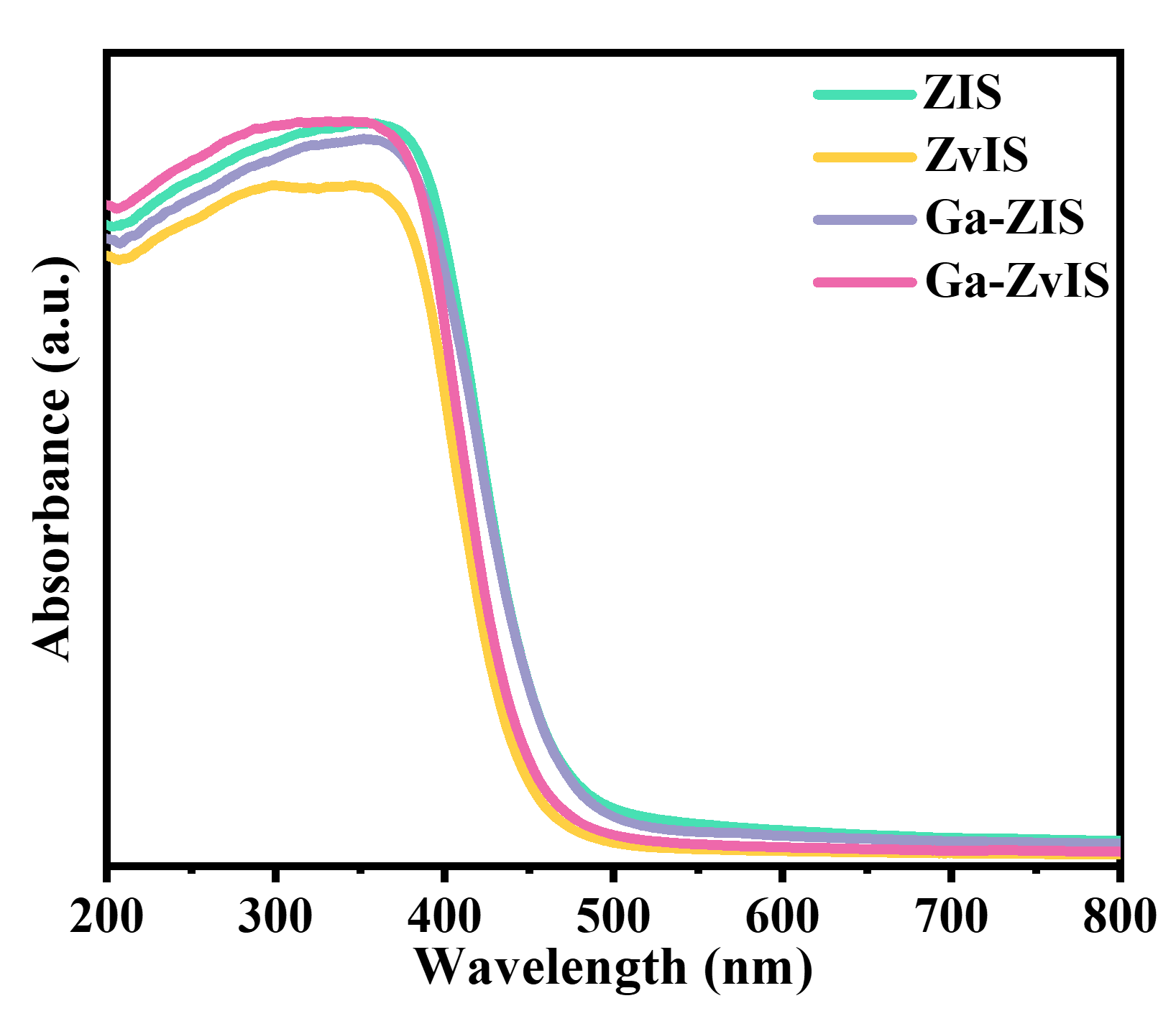


**Figure S15** Ultraviolet-visible absorption spectrum of samples


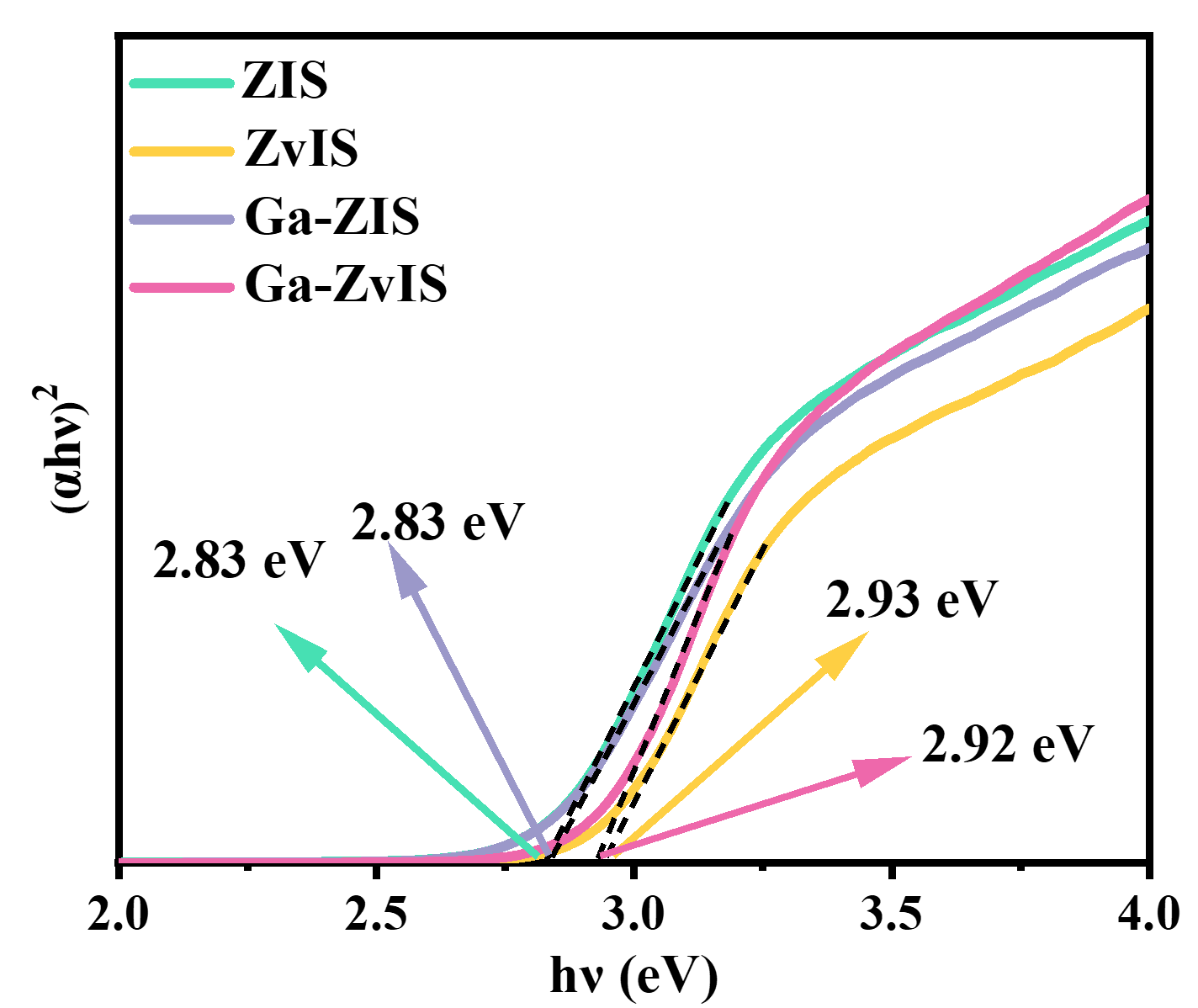


**Figure S16** Tauc plots of samples.


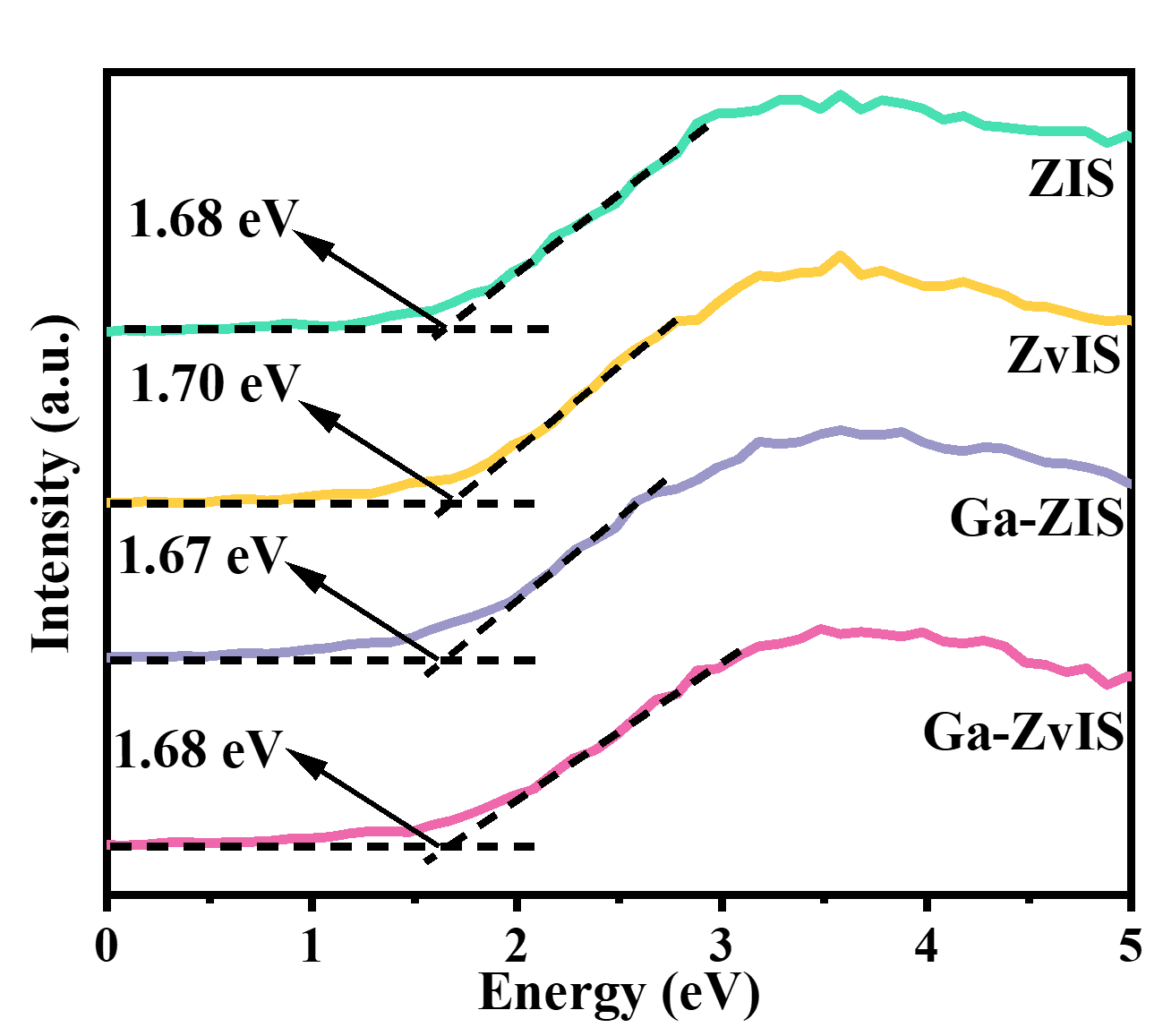


**Figure S17** Valence bands (VBs) spectra of samples from XPS


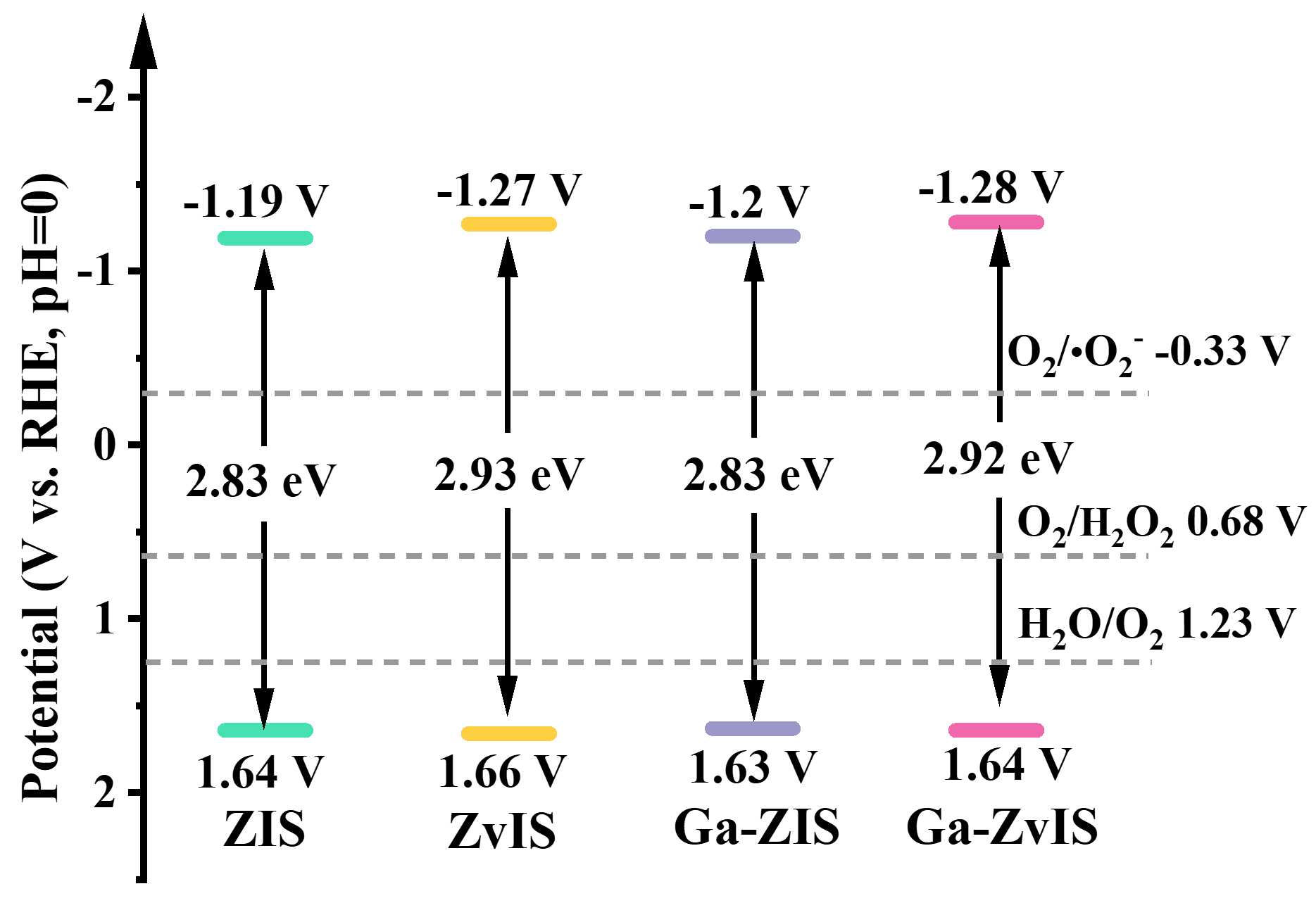


**Figure S18** Energy band structures of as-prepared samples


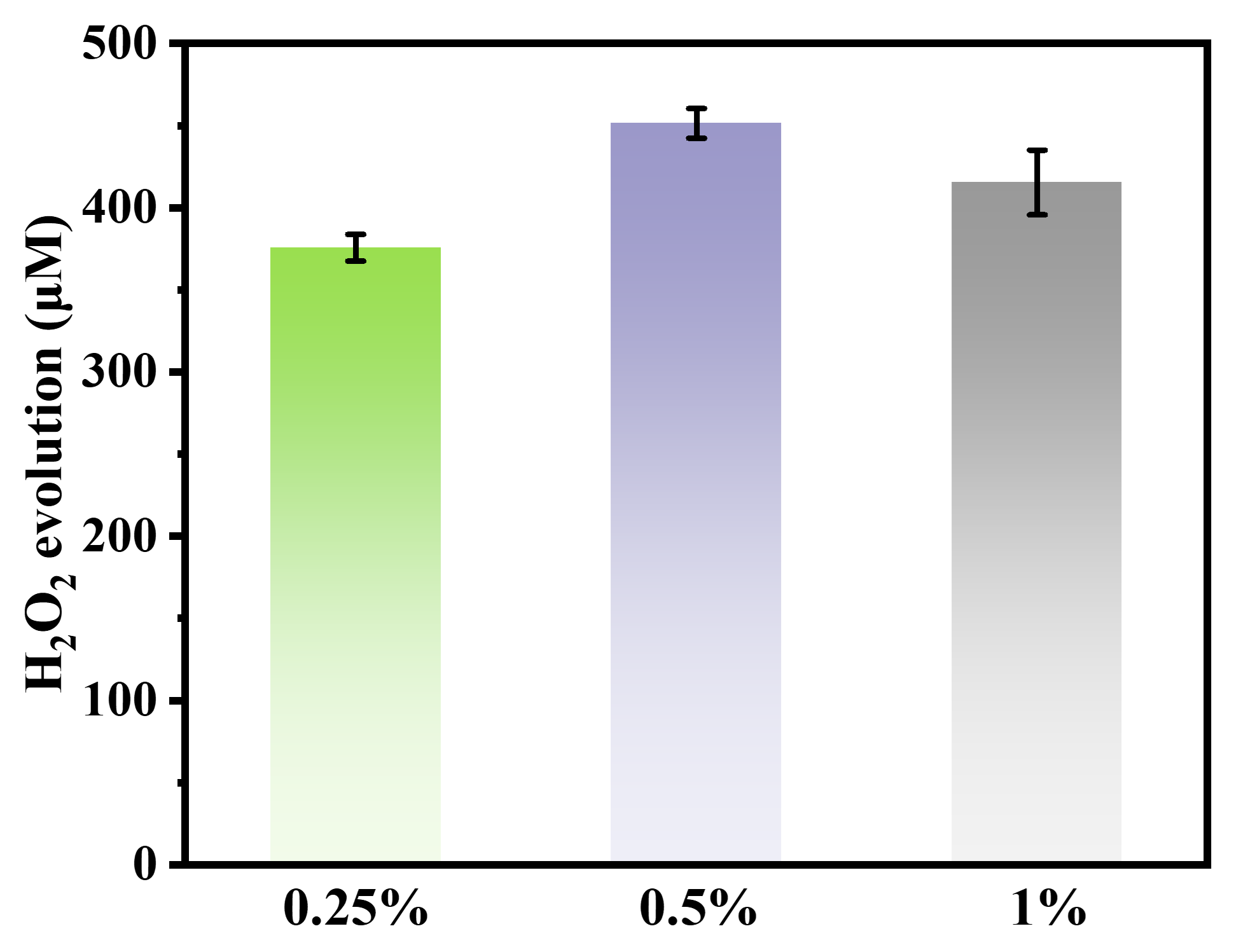


**Figure S19** Photocatalytic H_2_O_2_ evolution rates of different amounts of Ga.


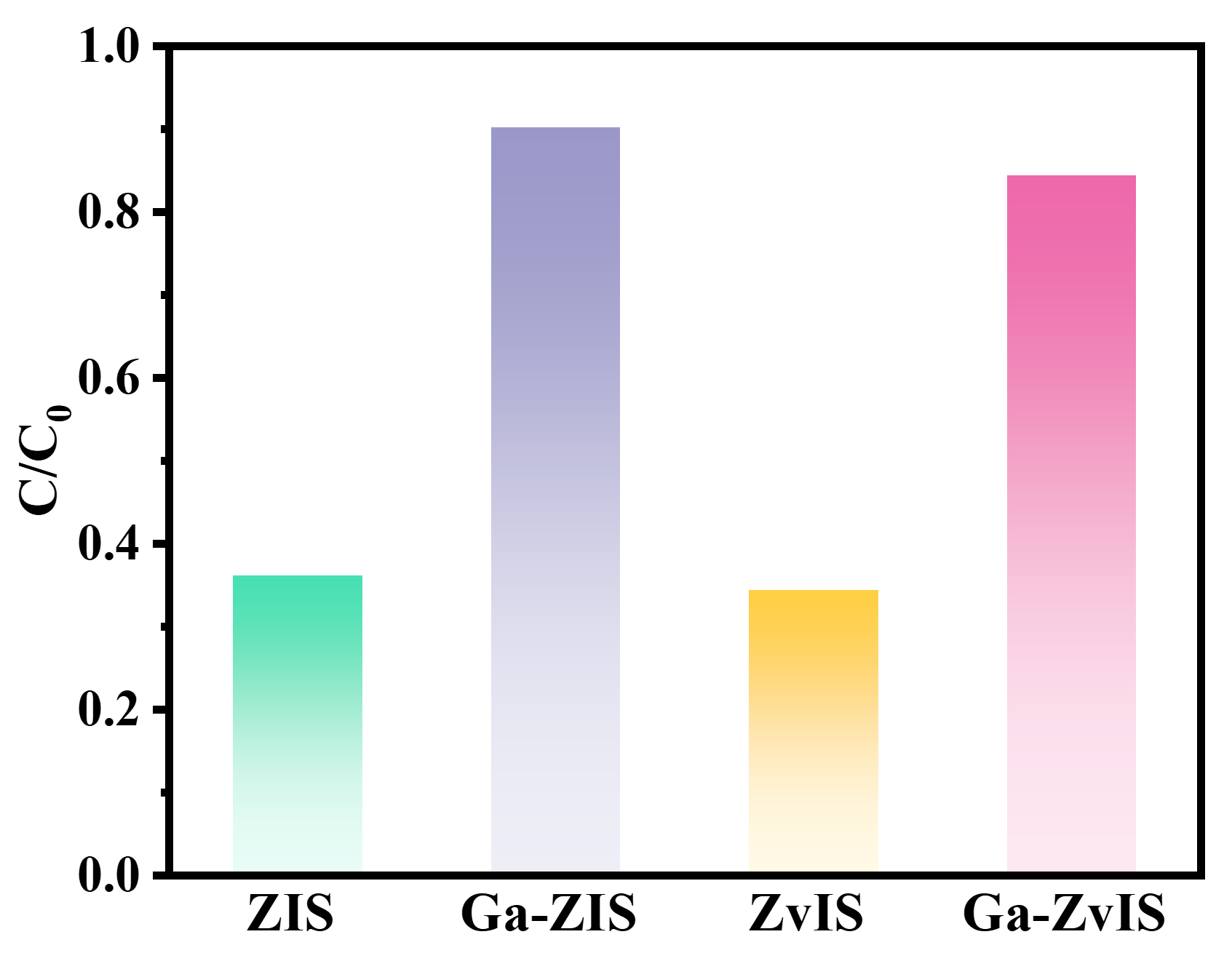


**Figure S20** Decomposition of H_2_O_2_ toward as-prepared catalysts.


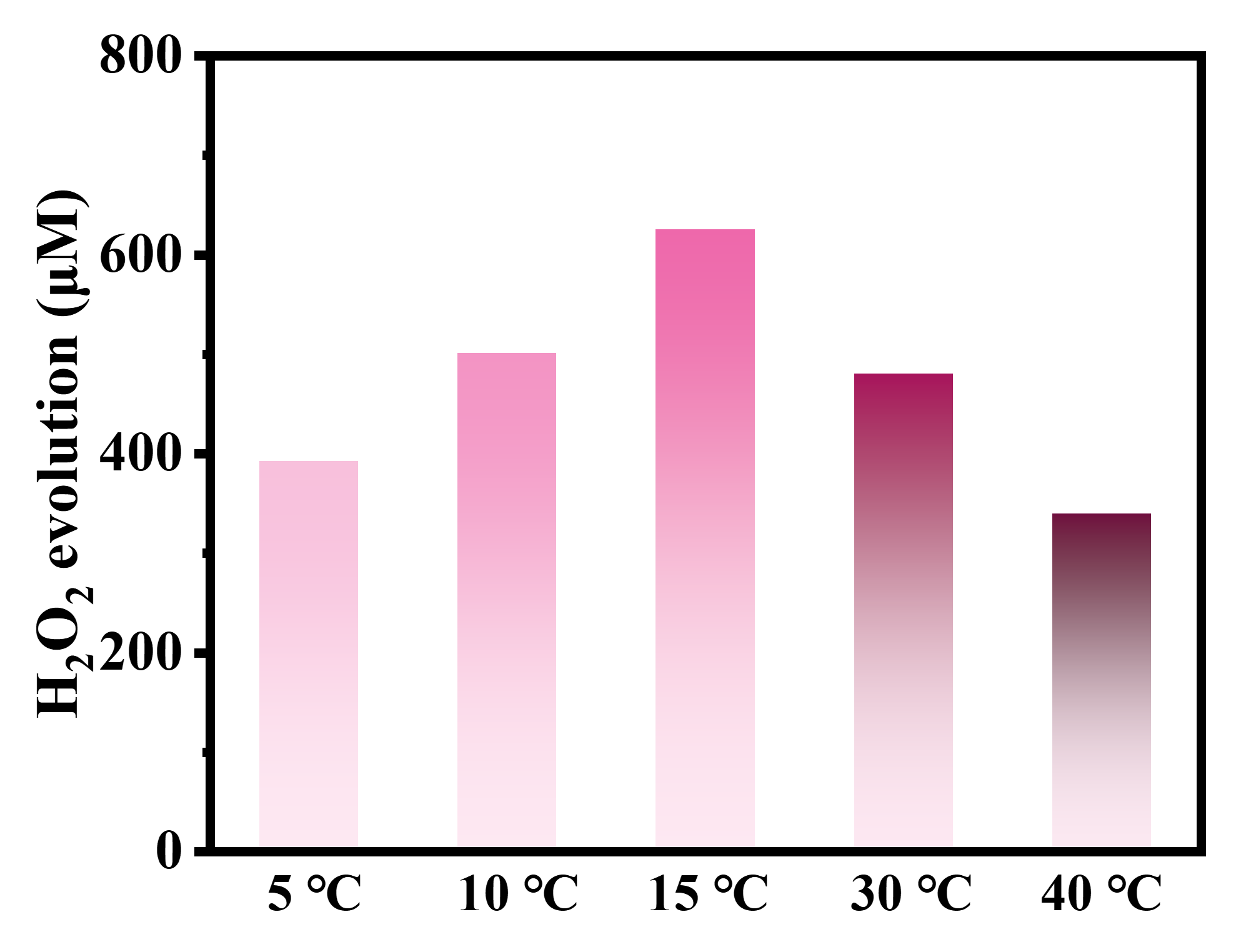


**Figure S21** Photocatalytic H_2_O_2_ evolution rates of Ga-ZvIS under different temperatures.


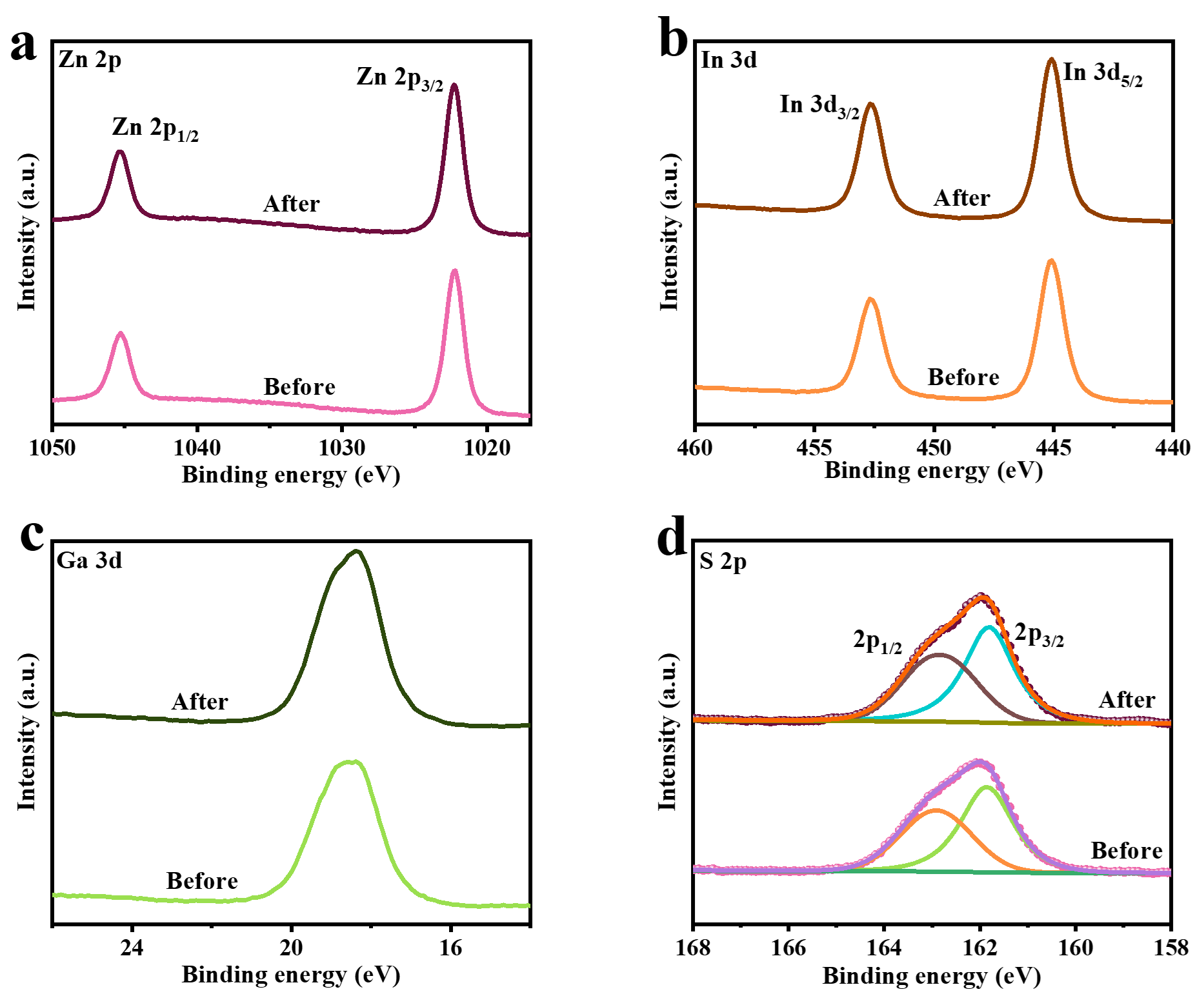


**Figure S22** (a) Zn 2p, (b) In 3d, (c) Ga 3d and (d) S 2p XPS spectra of Ga-ZvIS before and after Photocatalytic H_2_O_2_ evolution test


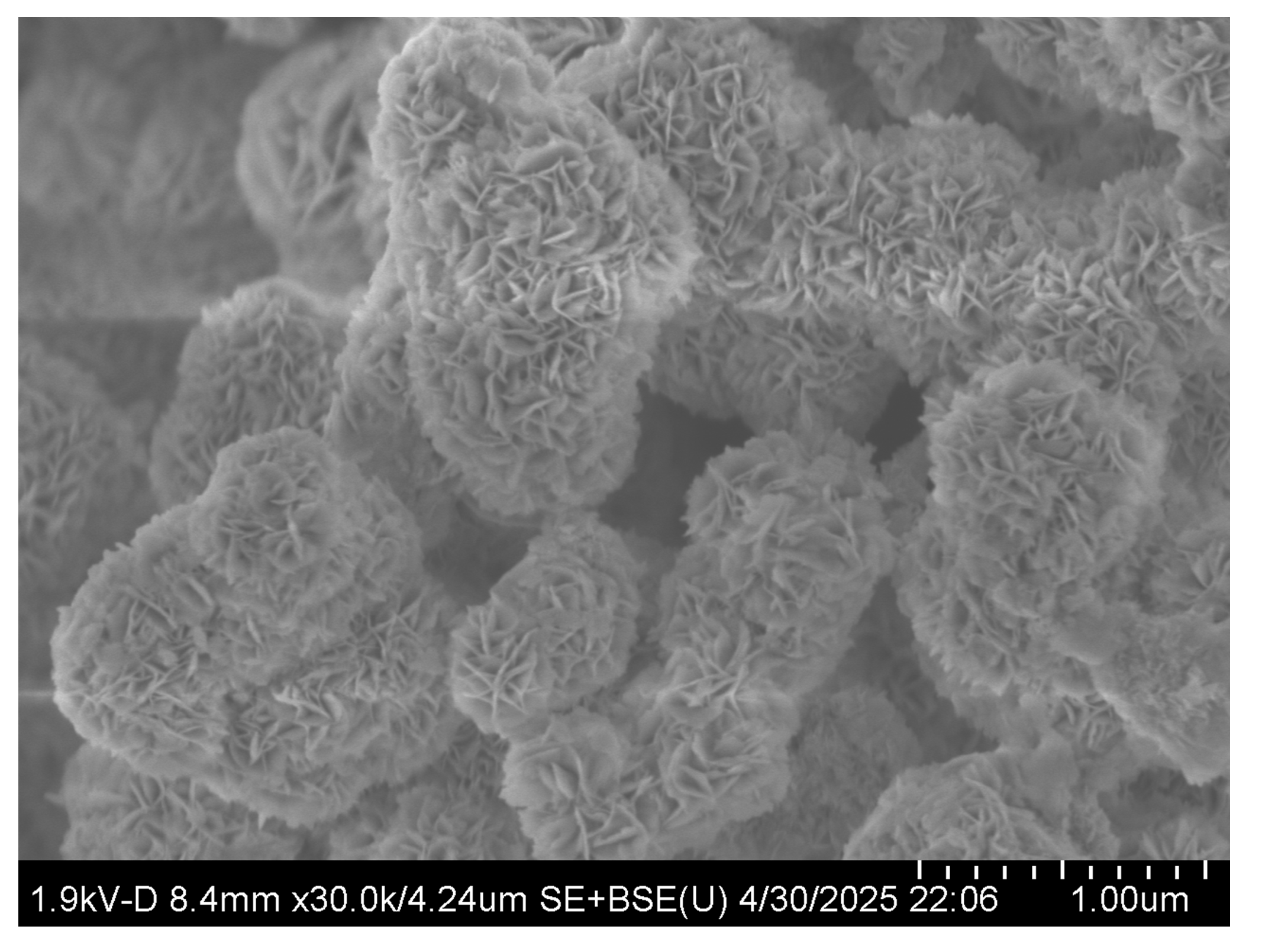


**Figure S23** SEM image of Ga-ZvIS after photocatalytic H_2_O_2_ evolution test


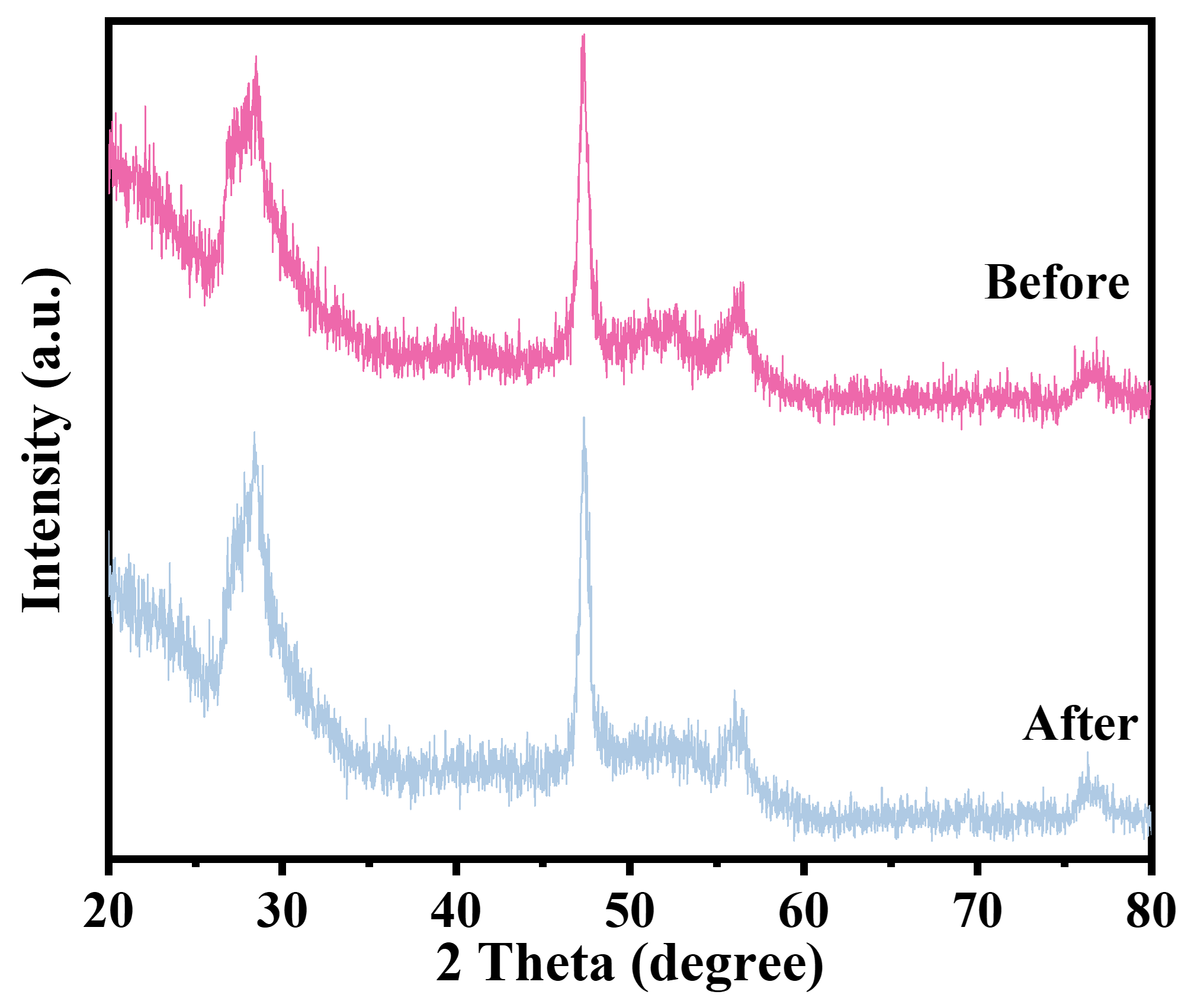


**Figure S24** XRD of Ga-ZvIS before and after photocatalytic H_2_O_2_ evolution test


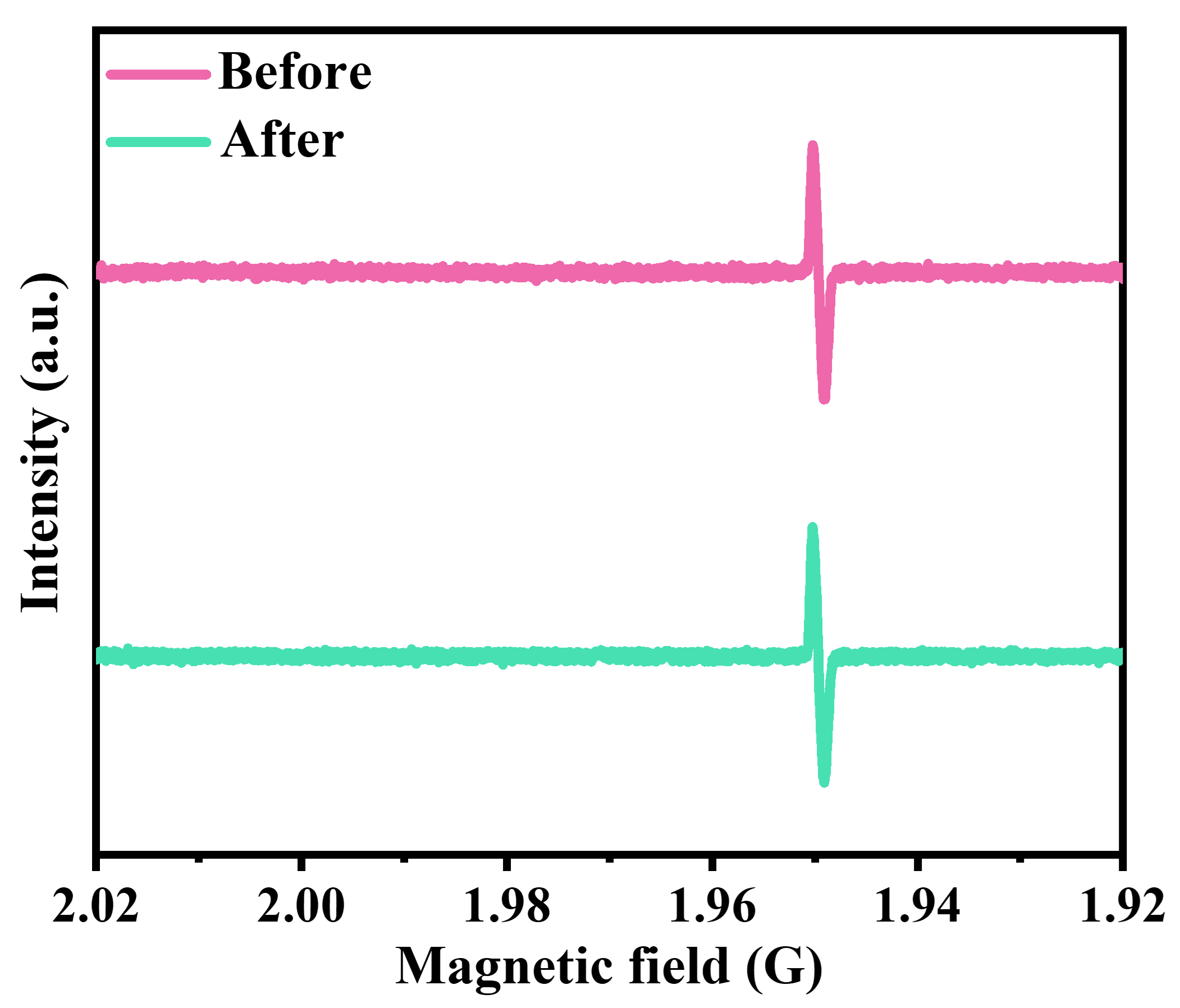


**Figure S25** ESR of Ga-ZvIS before and after photocatalytic H_2_O_2_ evolution test


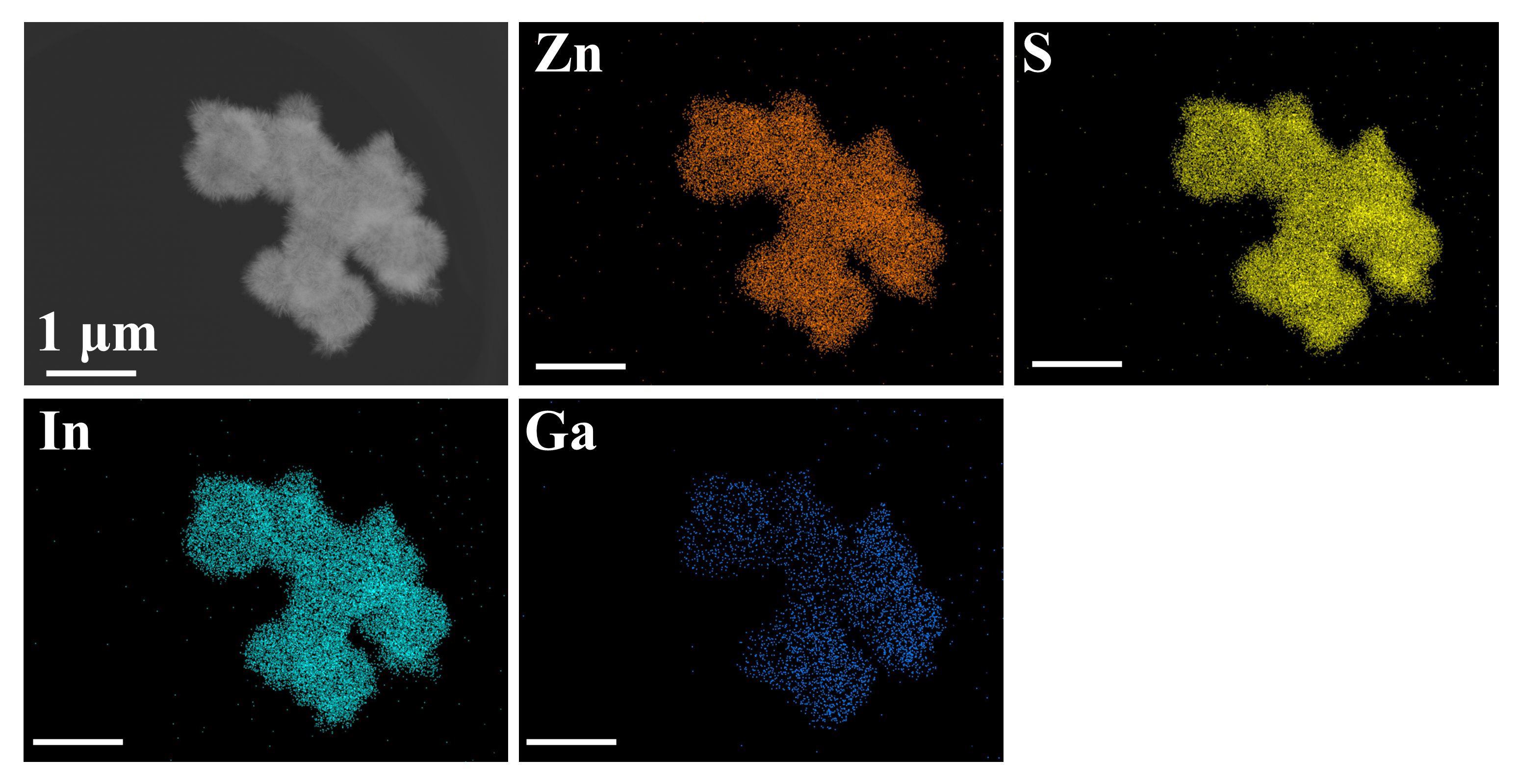


**Figure S26** EDS mapping images of Ga-ZvIS after photocatalytic H_2_O_2_ evolution test


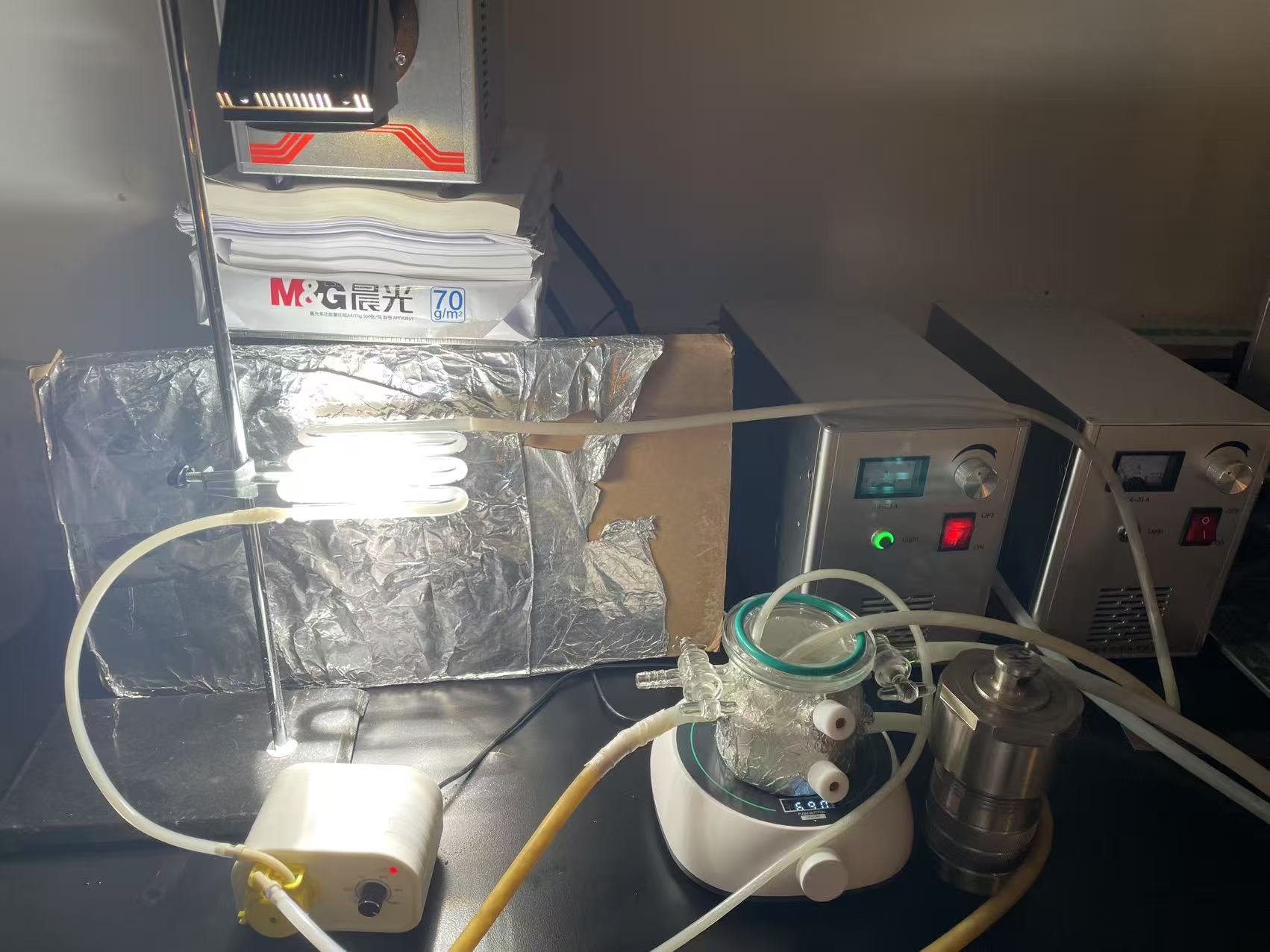


**Figure S27** Optical photographs of a flow catalytic device for photocatalytic production of H_2_O_2_


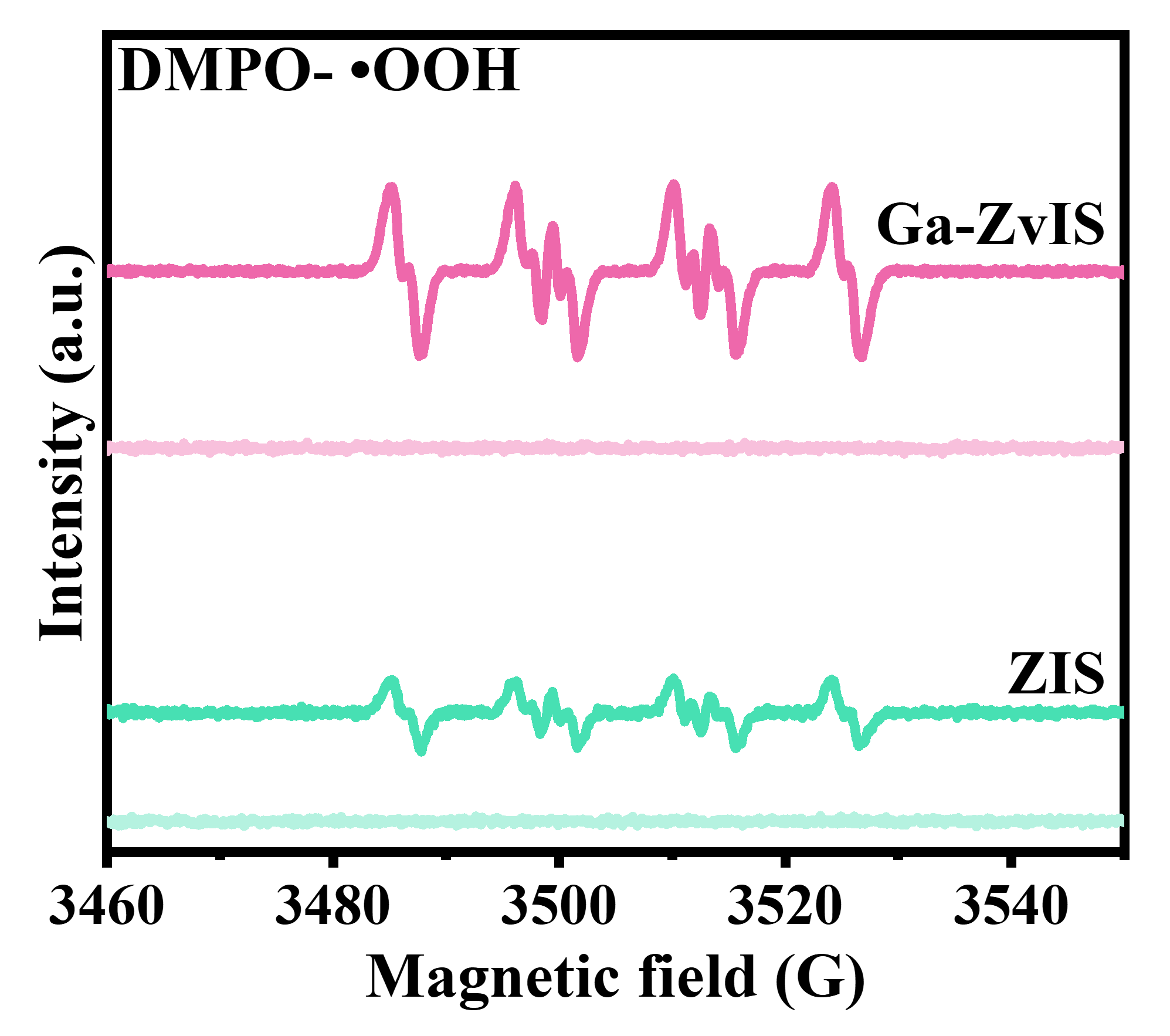


**Figure S28** DMPO spin-trapping ESR spectra for •OOH


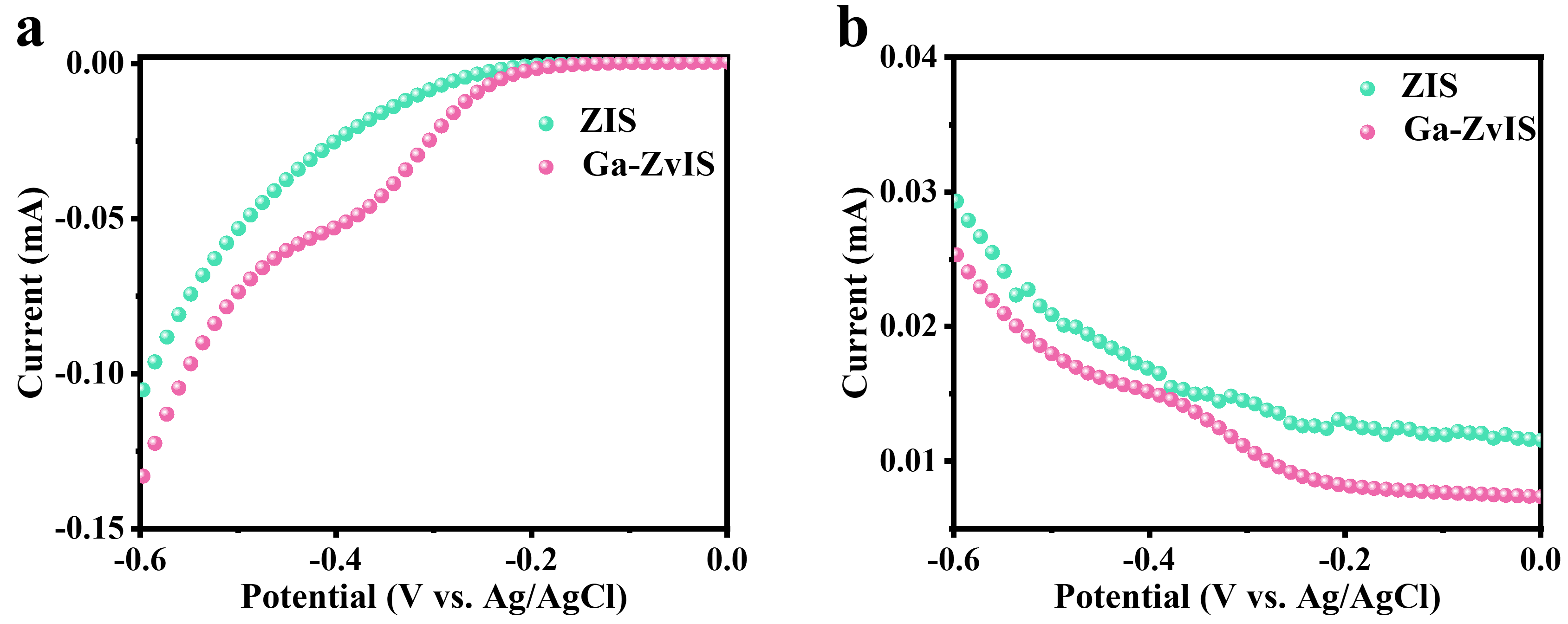


**Figure S29** The RRDE polarization curves of ZIS and Ga-ZvIS under O_2_ atmosphere (a) Disk current and (b) Ring current


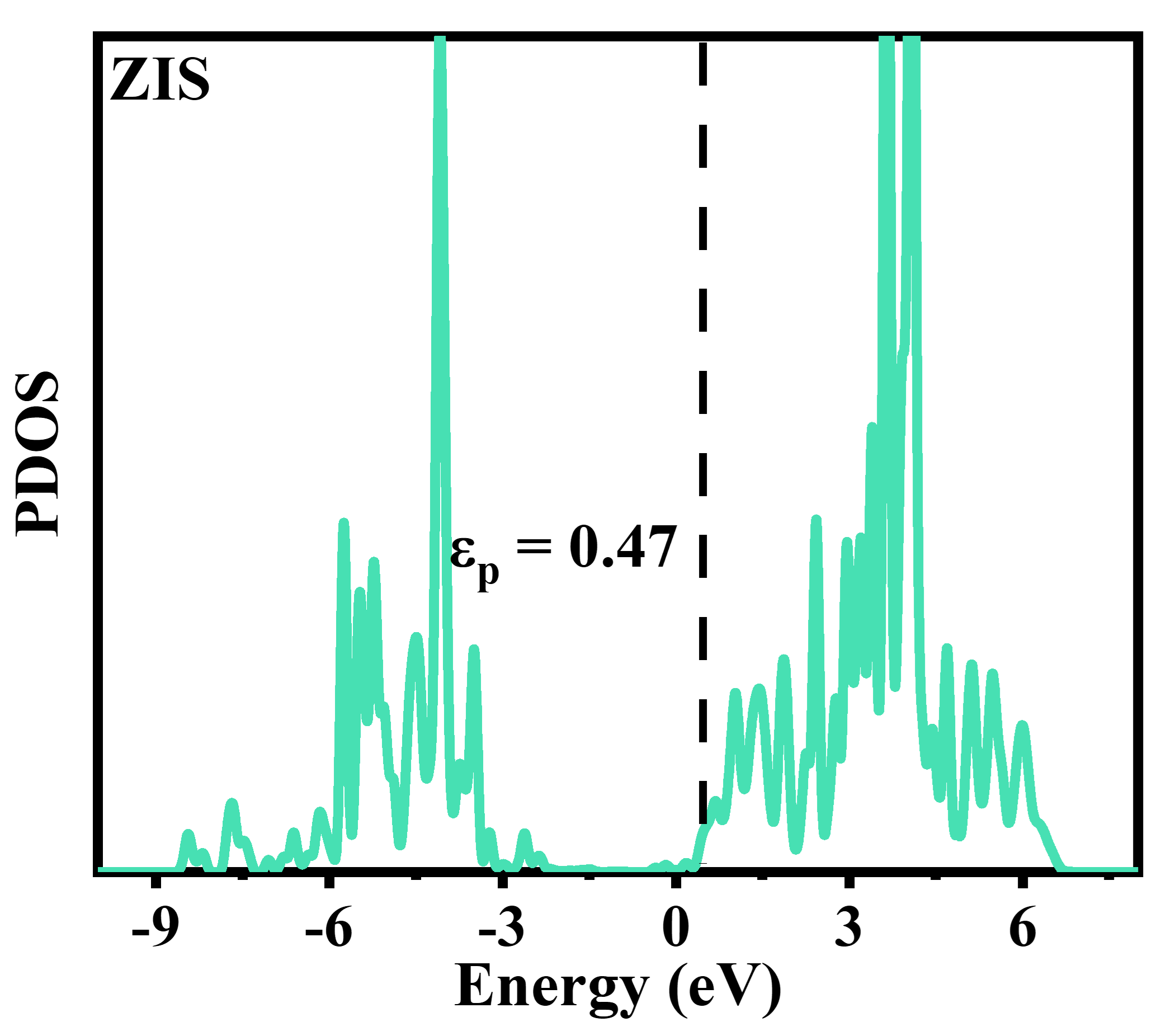


**Figure S30** The p-band centre of indium in ZIS


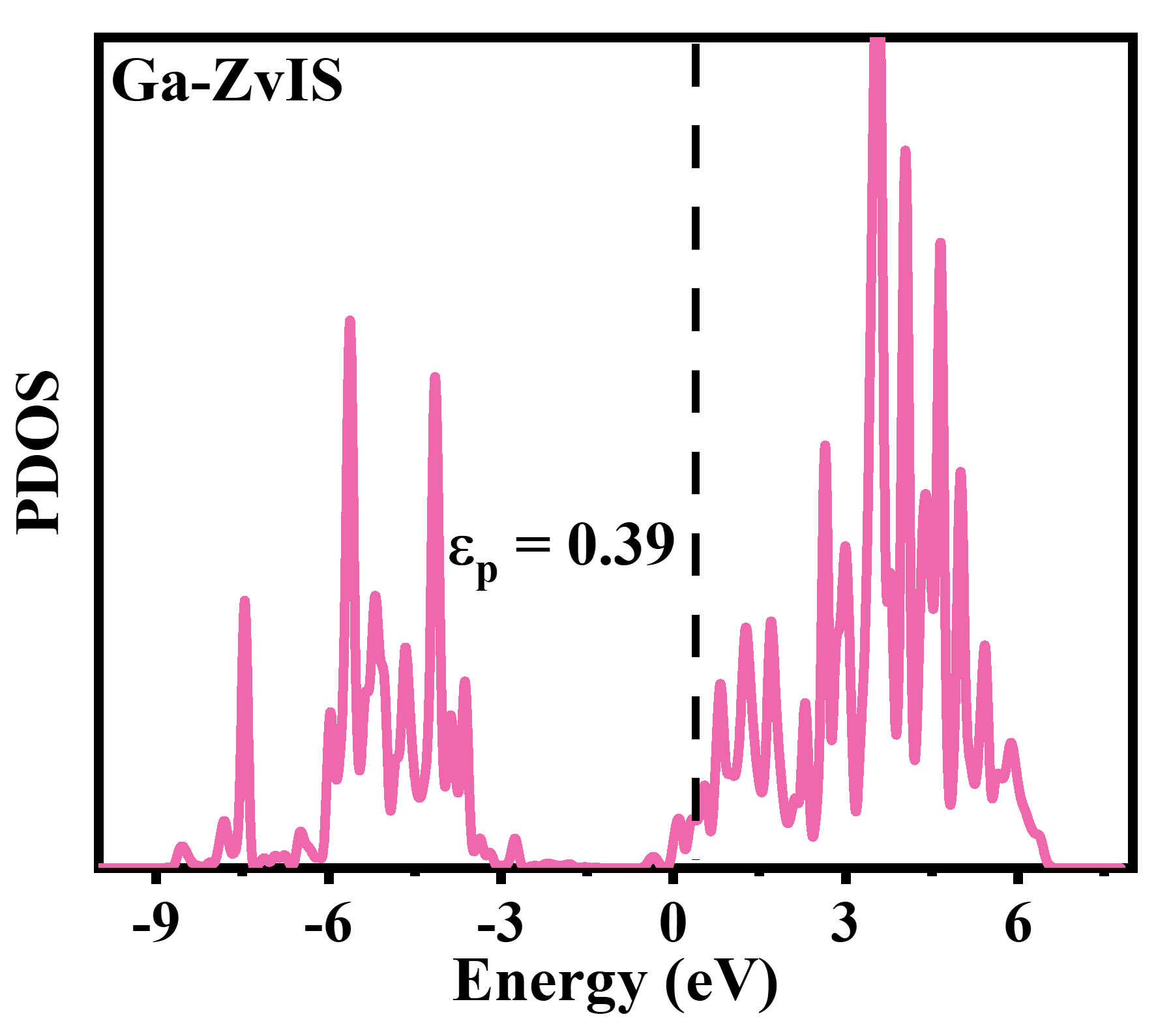


**Figure S31** The p-band centre of indium in Ga-ZvIS


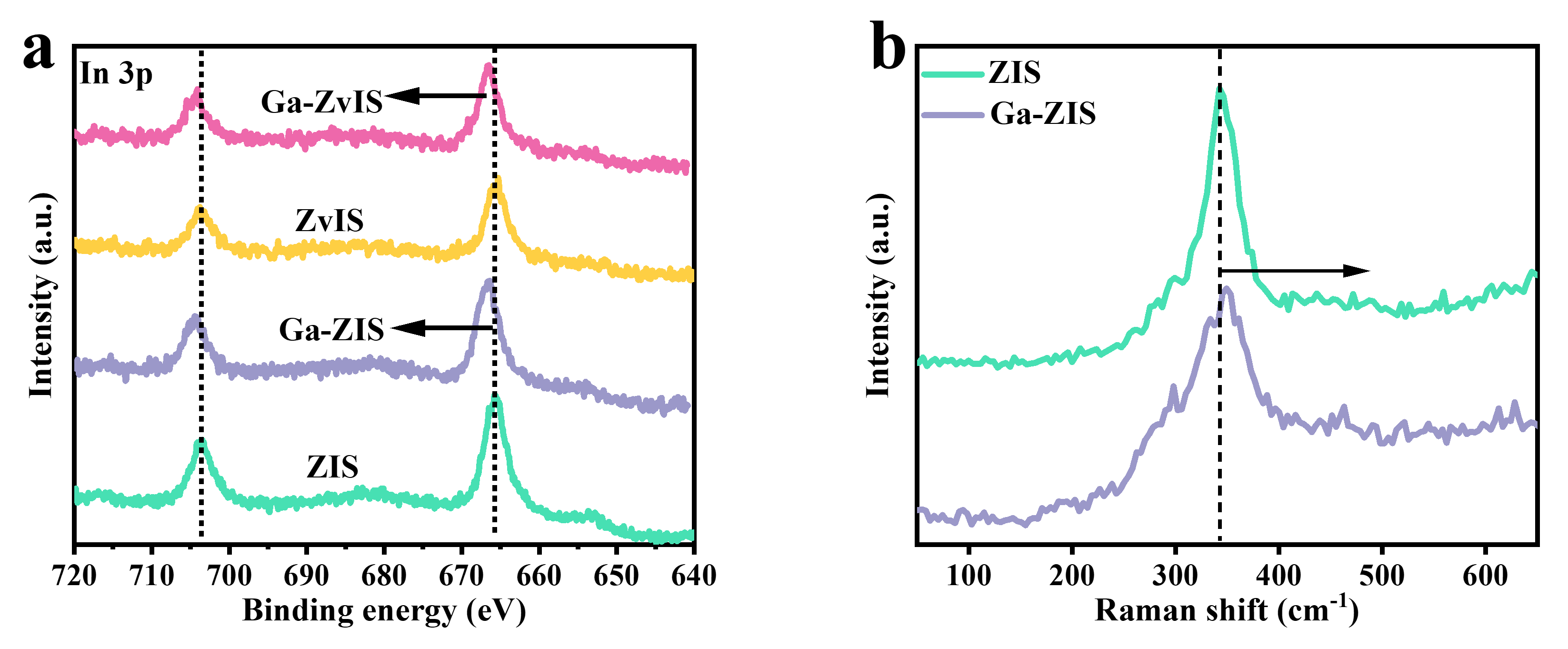


**Figure S32** (a) In 3p XPS of samples, (b) Raman spectroscopy of ZIS and Ga-ZIS

1. **Table. S1-S2**

**Table S1** Elemental content of ZIS and Ga-ZvIS from ICP.

| Sampls | In | Zn | S | Ga |
| --- | --- | --- | --- | --- |
| ZIS | 37.08 % | 32.26 % | 30.66 % | 0 |
| Ga-ZvIS | 36.85 % | 32.08 % | 30.79 % | 0.28 % |

**Table S2** Comparison of the performance with photocatalysts for photocatalytic production of H_2_O_2_ (with sacrificial agent).

| **Photocatalysts** | **Light source** | **Sacrificial agent** | **Yield (μmol·g^−1^·min^−1^**) | | **Ref.** |
| --- | --- | --- | --- | --- | --- |
| **Inorganic photocatalysts** | | | | | |
| **Ga-ZvIS** | AM 1.5G | **IPA** | | **187.8** | **This work** |
| P_2_ZIS | λ≥420 nm | IPA | | 35.1 | ^8^ |
| Nv–C≡N–CN | λ≥420 nm | IPA | | 51.6 | ^9^ |
| TF50-COF | λ≥400 nm | EtOH | | 29 | ^10^ |
| ZIS-0.7% | λ≥420 nm | IPA | | 51.4 | ^11^ |
| CNIO-GaSA | λ≥420 nm | EtOH | | 5.5 | ^12^ |
| CN/Zn-MOF(lc)/400  BCN  JNM-9-Ag  Cu(Ⅰ)-SA/WO_3_  C_3_N_5_ | λ≥420 nm  λ≥420 nm  λ≥420 nm  λ≥400 nm  λ≥420 nm | EtOH  EtOH  BA  Urea  EtOH | | 79.7  42.5  68.2  34  63.5 | ^13^  ^14^  ^15^  ^16^  ^17^ |
| O-Ru-ZIS | AM 1.5G | EtOH+IPA | | 159.5 | ^18^ |
| ZrS_1-y_S_2-x_ | AM 1.5G | BA | | 26 | ^19^ |
| PCN-Sb | λ≥400 nm | EtOH | | 160.5 | ^20^ |
| CN/Zn-OAc | λ≥400 nm | EtOH | | 129.2 | ^21^ |
| TiO_2_/Bi_2_O_3_ | 780≥λ≥350 nm | FA | | 48 | ^22^ |
| ZnO/CuInS_2_ | 1100≥λ≥350 nm | glycerin | | 22 | ^23^ |
| g-C_3_N_4_/NiS@Au | λ=420 nm | EtOH | | 88.3 | ^24^ |
| TF50-COF | λ≥400 nm | ETOH | | 28.9 | ^25^ |

**11. References**

1. G. Kresse and J. Furthmüller, *Phys. Rev. B*, 1996, 54, 11169–11186.

2. P. E. Blöchl, *Phys. Rev. B*, 1994, 50, 17953–17979.

3. G. Kresse and D. Joubert, *Phys. Rev. B*, 1999, 59, 1758–1775.

4. J. P. Perdew, K. Burke and M. Ernzerhof, *Phys. Rev. Lett.*, 1996, 77, 3865–3868.

5. S. Grimme, *J. Comput. Chem.*, 2006, 27, 1787–1799.

6. J. K. Nørskov, J. Rossmeisl, A. Logadottir, L. Lindqvist, J. R. Kitchin, T. Bligaard and H. Jónsson, *J. Phys. Chem. B*, 2004, 108, 17886–17892.

7. A. A. Peterson, F. Abild-Pedersen, F. Studt, J. Rossmeisl and J. K. Nørskov, *Energy Environ. Sci.*, 2010, 3, 1311–1315.

8. K. Zhang, L. Tian, J. Yang, F. Wu, L. Wang, H. Tang and Z.-Q. Liu, *Angew. Chem. Int. Ed.*, 2024, 63, e202317816.

9. X. Zhang, P. Ma, C. Wang, L. Gan, X. Chen, P. Zhang, Y. Wang, H. Li, L. Wang, X. Zhou and K. Zheng, *Energy Environ. Sci.*, 2022, 15, 830–842.

10. H. Wang, C. Yang, F. Chen, G. Zheng and Q. Han, *Angew. Chem. Int. Ed.*, 2022, 61, e202202328.

11. K.-L. Zhang, H.-C. Chen, L. Wang, H. Tang and Z.-Q. Liu, *Sci. Bull.*, 2025, 70, 536–545.

12. H. Tan, P. Zhou, M. Liu, Q. Zhang, F. Liu, H. Guo, Y. Zhou, Y. Chen, L. Zeng, L. Gu, Z. Zheng, M. Tong and S. Guo, *Nat. Synth*, 2023, 2, 557–563.

13. Y. Li, Y. Guo, D. Luan, X. Gu and X. W. Lou, *Angew. Chem. Int. Ed.*, 2023, 62, e202310847.

14. L. Ma, Y. Gao, B. Wei, L. Huang, N. Zhang, Q. Weng, L. Zhang, S. F. Liu and R. Jiang, *ACS Catal.*, 2024, 14, 2775–2786.

15. R.-Q. Xia, Z.-N. Liu, Y.-Y. Tang, T. Wu, X. Luo, Q.-M. Deng, G.-H. Ning and D. Li, *Angew. Chem. Int. Ed.*, 2025, 64, e202514091.

16. F. Yang, C. Feng, S. Zuo, Q. Wang, F. Wei, M. Hu, Y. Ren, D. Liu, W.-L. Li, S. Wang, H. S. Alqahtani, Y. H. Ng and H. Zhang, *J. Am. Chem. Soc.*, 2025, 147, 17112–17120.

17. Z. Li, Y. Zhou, Y. Zhou, K. Wang, Y. Yun, S. Chen, W. Jiao, L. Chen, B. Zou and M. Zhu, *Nat. Commun.*, 2023, 14, 5742.

18. C. Ding, S. Zhao, X. Ruan, D. Jiao, M. Xu, G. Fang, D. Meng, W. Zhang, J. Leng, Z. Jiang, L. Zhang, S. K. Ravi, S. Zhan and X. Cui, *Adv. Mater.*, 2025, n/a, e09867.

19. Z. Tian, C. Han, Y. Zhao, W. Dai, X. Lian, Y. Wang, Y. Zheng, Y. Shi, X. Pan, Z. Huang, H. Li and W. Chen, *Nat. Commun.* , 2021, 12, 2039.

20. H. Lu, H. Yin, J. Harmer, M. Xiao, J. You, P. Chen, T. Lin, A. Du, Z. Wang and L. Wang, *Angew. Chem. Int. Ed.*, 2025, 64, e202413769.

21. Y. Li, Y. Guo, G. Fan, D. Luan, X. Gu and X. W. Lou, *Angew. Chem. Int. Ed.*, 2024, 63, e202317572.

22. B. He, Z. Wang, P. Xiao, T. Chen, J. Yu and L. Zhang, *Adv. Mater.*, 2022, 34, 2203225.

23. K. Meng, J. Zhang, B. Cheng, X. Ren, Z. Xia, F. Xu, L. Zhang and J. Yu, *Adv. Mater.*, 2024, 36, 2406460.

24. W. Zhong, A. Meng, Y. Su, H. Yu, P. Han and J. Yu, *Angew. Chem. Int. Ed.*, 2025, 64, e202425038.

25. H. Wang, C. Yang, F. Chen, G. Zheng and Q. Han, *Angew. Chem. Int. Ed.* , 2022, 61, e202202328.
